# Tumor-infiltrating Leukocyte Profiling Defines Three Immune Subtypes of NSCLC with Distinct Signaling Pathways and Genetic Alterations

**DOI:** 10.1158/2767-9764.CRC-22-0415

**Published:** 2023-06-13

**Authors:** Kazunori Aoki, Yukari Nishito, Noriko Motoi, Yasuhito Arai, Nobuyoshi Hiraoka, Tatsuhiro Shibata, Yukiko Sonobe, Yoko Kayukawa, Eri Hashimoto, Mina Takahashi, Etsuko Fujii, Takashi Nishizawa, Hironori Fukuda, Kana Ohashi, Kosuke Arai, Yukihiro Mizoguchi, Yukihiro Yoshida, Shun-ichi Watanabe, Makiko Yamashita, Shigehisa Kitano, Hiromi Sakamoto, Yuki Nagata, Risa Mitsumori, Kouichi Ozaki, Shumpei Niida, Yae Kanai, Akiyoshi Hirayama, Tomoyoshi Soga, Toru Maruyama, Keisuke Tsukada, Nami Yabuki, Mei Shimada, Takehisa Kitazawa, Osamu Natori, Noriaki Sawada, Atsuhiko Kato, Teruhiko Yoshida, Kazuki Yasuda, Hideaki Mizuno, Hiroyuki Tsunoda, Atsushi Ochiai

**Affiliations:** 1 Department of Immune Medicine, National Cancer Center Research Institute, Chuo-ku, Tokyo, Japan.; 2 Kamakura Research Laboratories, Chugai Pharmaceutical Co., Ltd., Kamakura, Kanagawa, Japan.; 3 Department of Diagnostic Pathology, National Cancer Center Hospital, Chuo-ku, Tokyo, Japan.; 4 Divison of Cancer Genomics, National Cancer Center Research Institute, Chuo-ku, Tokyo, Japan.; 5 Department of Analytical Pathology, National Cancer Center Research Institute, Chuo-ku, Tokyo, Japan.; 6 Department of Thoracic Surgery, National Cancer Center Hospital, Chuo-ku, Tokyo, Japan.; 7 Advanced Medical Development Center, Cancer Research Hospital, Japanese Foundation for Cancer Research, Koto-ku, Tokyo, Japan.; 8 Department of Clinical Genomics, National Cancer Center Research Institute, Chuo-ku, Tokyo, Japan.; 9 Medical Genome Center, Research Institute, National Center for Geriatrics and Gerontology, Obu, Aichi, Japan.; 10 Bioresource Research Center, Graduate School of Medical and Dental Science, Tokyo Medical and Dental University, Bunkyo-ku, Tokyo, Japan.; 11 Department of Pathology, School of Medicine, Keio University, Sinjyuku-ku, Tokyo, Japan.; 12 Institute for Advanced Biosciences, Keio University Tsuruoka, Yamagata, Japan.; 13 Department of Genetic Medicine and Services, National Cancer Center Hospital, Chuo-ku, Tokyo, Japan.; 14 National Center for Global Health and Medicine, Shinjuku-ku, Tokyo, Japan.; 15 Exploratory Oncology Research and Clinical Trial Center, National Cancer Center, Kashiwa, Chiba, Japan.

## Abstract

Resistance to immune checkpoint blockade remains challenging in patients with non–small cell lung cancer (NSCLC). Tumor-infiltrating leukocyte (TIL) quantity, composition, and activation status profoundly influence responsiveness to cancer immunotherapy. This study examined the immune landscape in the NSCLC tumor microenvironment by analyzing TIL profiles of 281 fresh resected NSCLC tissues. Unsupervised clustering based on numbers and percentages of 30 TIL types classified adenocarcinoma (LUAD) and squamous cell carcinoma (LUSQ) into the cold, myeloid cell–dominant, and CD8^+^ T cell–dominant subtypes. These were significantly correlated with patient prognosis; the myeloid cell subtype had worse outcomes than the others. Integrated genomic and transcriptomic analyses, including RNA sequencing, whole-exome sequencing, T-cell receptor repertoire, and metabolomics of tumor tissue, revealed that immune reaction–related signaling pathways were inactivated, while the glycolysis and K-ras signaling pathways activated in LUAD and LUSQ myeloid cell subtypes. Cases with *ALK* and *ROS1* fusion genes were enriched in the LUAD myeloid subtype, and the frequency of *TERT* copy-number variations was higher in LUSQ myeloid subtype than in the others. These classifications of NSCLC based on TIL status may be useful for developing personalized immune therapies for NSCLC.

**Significance:**

The precise TIL profiling classified NSCLC into novel three immune subtypes that correlates with patient outcome, identifying subtype-specific molecular pathways and genomic alterations that should play important roles in constructing subtype-specific immune tumor microenvironments. These classifications of NSCLC based on TIL status are useful for developing personalized immune therapies for NSCLC.

## Introduction

Lung cancer is the leading cause of cancer-related deaths worldwide (1). The emergence of immune checkpoint blockade (ICB) has transformed the therapeutic strategies for various cancers, including those for non–small cell lung cancer (NSCLC). However, clinical studies have revealed significant drug resistance; only 20% of patients with NSCLC respond to anti-PD-1/PD-L1 therapy (2). The primary reason for this reduced response is the immunosuppressive tumor microenvironment (TME), which is generated by several factors, including the lack of tumor-infiltrating leukocytes (TIL) or the presence of immunosuppressive factors (3). Because these TME characteristics are different in each case (4), it is important to develop specialized immunotherapies suitable for each immune vulnerability.

TIL quantity, composition, and activation status profoundly influence responsiveness to cancer immunotherapy (4). While the malignant components of NSCLC have been profiled at the molecular level, including mutational spectra and other molecular features (5, 6), it is unclear whether molecular subtyping reflects TME immune conditions. While the immune cell contents of NSCLC and other solid cancers have been profiled using transcriptional signatures (7), this method has not been conclusively shown to represent actual immune cell components. Therefore, we assessed 30 types of immune cells present in tumor tissue using flow cytometry (FCM) to better profile immune functional status and identify predominant TME cell types, to guide better decision-making.

Although deciphering the immune TME can improve the tailoring of immunotherapy, integrated genomic and transcriptomic analyses are uncommon. Therefore, in this study, for the first time, we constructed a database from FCM, RNA sequencing (RNA-seq), whole-exome sequencing (WES), T-cell receptor (TCR) repertoires, and metabolomic data accompanied by clinicopathologic findings; this combination offers a multifaceted view of the immune TME.

## Materials and Methods

### Patients and Samples

We enrolled 281 patients with NSCLC (adenocarcinoma, *n* = 155; squamous cell carcinoma, *n* = 80; other types including small cell cancer, large cell cancer, sarcoma, neuroendocrine tumor, adenosquamous carcinoma, pleiomorphic carcinoma, adenoid cystic carcinoma, *n* = 46) who underwent surgical resections at the National Cancer Center Hospital Japan (2017–2019). Patient characteristics are summarized in Supplementary Table S1. All patients provided written informed consent before sampling, and the study abided by the principles of the Declaration of Helsinki. This study was approved (2016-124) by the National Cancer Center Ethics Committee. The patients who did not receive preoperative treatment, such as neoadjuvant chemotherapy, were included in this study. As clinicopathologic factors, age at surgery, gender, pathologic tumor–node–metastasis stage (8th edition), histologic type and subtype based on the fourth edition of World Health Organization (WHO) classification, tumor differentiation, lymphatic (ly), venous (v), and pleural invasions (pl), intra-pulmonary metastasis (pm), tumor spread through air spaces (STAS), and background lung disease (emphysema, pulmonary fibrosis) were collected from the medical chart. As histopathologic factors, tertiary lymphoid structure (TLS) grade, degree of inflammatory cell infiltrates (lymphocyte, neutrophil, macrophage), were semiquantitatively evaluated using tissue microarrays (TMA) of surgically resected specimens. The number of TLS per overall sampled tissue (2-mm-core circled area = 3.14 mm^2^) was scored on a three-tiered scale: 0, none of lymphoid structure; 1, 1–2 of lymphoid structures; 2, more than 3 of lymphoid structures. The degree of inflammatory cell infiltrates scored into three-tiered relative categories: none-mild/moderate/severe.

### Tissue Samples

Tumor and histologically normal tissue adjacent to the tumor (NAT) samples were received within 2 hours after resection and immediately dissociated into single cells by mincing and incubated in RPMI1640 supplemented with 80 U/mL DNase I (Merck), 300 U/mL collagenase I (Merck), and 60 U/mL hyaluronidase IV (Merck) at 37°C for 30 minutes using a gentleMACS Octo Dissociator with heaters (Miltenyi Biotec). The single-cell suspension was washed, passed through 40-μm cell strainers, suspended in Bambanker freezing medium (Nippon Genetics), and stored at −150 °C until FCM.

### FCM

Cryopreserved cells were thawed and incubated with Fixable Viability Dye eFluor 506 (Thermo Fisher Scientific) for dead cell staining for 30 minutes at 4°C and incubated with FcR blocking reagent (Miltenyi Biotec) for 10 minutes at 4°C, followed by staining with mAbs (Supplementary Table S2) for 30 minutes at 4°C. For Foxp3 transcription factor staining, cells were fixed and permeabilized using a staining buffer set (Thermo Fisher Scientific) and incubated with mAb for 1 hour at 4°C. After washing, data were acquired on a FACSymphony A5 instrument and analyzed using FlowJo software (both from BD Biosciences, RRID: SCR 008520).

### Immune Profiling Data Clustering

To identify subtypes reflecting the quantities and frequencies of each immune cell type, cell proportions per CD45^+^ cells (%CD45) and log_10_-transformed cell count per gram of tumor + 1 (cell density) were scaled across patients and then combined. Unsupervised hierarchical clustering was conducted with the Ward.D2 algorithm, and Spearman correlation distance was analyzed using the R “hclust” package.

### RNA-seq

Total RNA was extracted from whole-tumor tissues using an RNeasy Mini Kit (QIAGEN) following the manufacturer's instructions. RNA integrity was evaluated with TapeStation (Agilent Technologies). cDNA was prepared using the NEBNext Ultra Directional RNA Library Prep kit (New England Biolabs). Libraries were analyzed using Agilent 4200 TapeStation (Agilent Technologies) and then subjected to next-generation sequencing of 125-bp paired-end reads using the HiSeq PE Cluster Kit v4 cBot and HiSeq SBS Kit v4 with a Hiseq2500 platform (Illumina). Raw HiSeq data were converted to the FASTQ format with bcl2fastq (Illumina) and cleaned using QCleaner software (Amelieff) using the Resequence analysis pipeline (Amelieff). For expression profiling, paired-end reads were aligned to the hg38 human genome assembly using STAR, and count values were calculated using the STAR quant mode (8). The Transcripts Per Million (TPM) value for each RefSeq was calculated using RSEM with bowtie mapping (9). Fusion genes were detected using STAR-Fusion (10).

### Expression and Gene Set Enrichment Analyses

Differentially expressed genes were identified by the Wald test using the R “DESeq2” package; genes with base mean values >100 were used for later analysis. Gene set enrichment analysis (GSEA, RRID: SCR 003199) was performed using a hallmark gene set from the ClusterProfiler (RRID:SCR_016884) R package and Gene Ontology (GO) Biological Process ontology using the “msigdbr” package. Hierarchical clustering was conducted using the z-scores of log_2_ (TPM+1) expression values.

To assign molecular subtypes, TPM+1 values were log transformed and gene median centered. For each sample, Pearson correlations were calculated with molecular subtype predictor centroids for adenocarcinoma (LUAD; ref. 11) and squamous cell carcinoma (LUSQ; ref. 12). The centroid allowed molecular subtype prediction with the largest correlation value.

The steps of the cancer-immunity cycle were described using eight axes of the immunogram score (IGS): IGS1, T-cell immunity; IGS2, tumor antigenicity; IGS3, priming and activation; IGS4, trafficking and infiltration; IGS5, recognition of tumor cells; IGS6, inhibitor cells; IGS7, checkpoint expression; and IGS8, inhibitory molecules (13). As described in the original article, IGSs were calculated, but IGS2 was not examined because neoantigens were not included in our analyses. Antitumor immune reactivity was scored as a higher number (toward 5), while immunosuppressive reactivity was scored as a lower number (toward 0). Accordingly, higher signatures were plotted as higher scores in IGS1–5, whereas higher signatures were plotted as lower scores in IGS6–8 in radar plots.

### IHC

Formalin-fixed and paraffin-embedded (FFPE) lung cancer tissues, 65 LUAD and 44 LUSQ, were obtained from the National Cancer Center Biobank, Japan. Using FFPE blocks, TMAs were constructed from two representative cores per tumor. The core was 2 mm in diameter. Immunostaining for CD8, FOXP3, CD20, Ki67, and CD33 on 4-μm-thick TMA sections was performed using the autostainer DAKO LINK48 (Agilent Technologies) according to the manufacturer's protocol. All stained slides were scanned at 40× resolution using Nanozoomer (Hamamatsu Photonics). For CD8, FOXP3, CD20, and Ki67, the digital images were analyzed using the HALO system (v3.1, Indica Labs) with the use of the CytoNuclear Algorithm v2.0.5. The image zoom for the HALO analysis was set at 0.166 for CD8 and CD20 and 0.205 for Foxp3 and Ki67. The positivity of each marker was represented by the number of total positive cells within each tissue core. For CD33, the digital images were analyzed using the HALO system (v3.5) with the Area Quantification v2.4. Because myeloid cells have a diverse cell morphology, it is difficult to recognize one cell of myeloid cell lineage by the Halo system. Therefore, the proportion of CD33-positive staining area per examined tissue area (percentage, %; range 0–100) was calculated to analyze myeloid lineage cells on the tissue specimen. Tissue area included both tumor and non-tumor areas, and blank areas were excluded. All output images were reviewed by pathologists (E. Fujii, A. Kato, N. Motoi) to ensure the consistency of the positivity with the actual images. Primary antibodies and staining conditions are listed in Supplementary Table S3.

### WES and Mutation Analysis

DNA was extracted using the QIAamp DNA Mini Kit (QIAGEN), and libraries were prepared for WES using the NEBNext Ultra DNA Library Prep Kit (New England Biolabs) following the manufacturer's instructions. Adaptor-ligated samples were amplified using six PCR cycles. The amplified DNA fragments were enriched for exotic fragmentations using the SureSelect Human All Exon Kit v6 (Agilent Technologies). Massively parallel sequencing was performed using the HiSeq2500 platform (Illumina). Mutation calling was performed using the best-practice workflow with GATK v4.0.8.1 (RRID: SCR 001876). Paired-end WES reads were independently aligned to the human reference genome (hg38) using BWA-MEM (RRID: SCR 017619). Somatic mutations were identified using Mutect2 and annotated using SnpEff (14). CNVkit was used to estimate the log_2_ copy ratios (15) in R-3.6.1.

### TCR Repertoire Analysis

Following the manufacturer's instructions, TCR sequencing libraries were prepared from tumor RNA using a QIAseq immune repertoire RNA library kit (QIAGEN). Specific cDNA libraries for TCRα, TCRβ, TCRγ, and TCRδ variable regions were constructed from 300 ng of total tumor RNAs and matched NATs using TCR constant region–specific and universal PCR primers. Unique molecular indices (UMI) were added before library amplification to reduce PCR bias and increase the accuracy of the assessment of repertoire diversity. TCR cDNA libraries were sequenced in paired-end mode with 251-bp reads using a MiSeq sequencer (Illumina). Sequencing data were analyzed using the IMSEQ. Reads were aligned to the reference V, D, and J regions of *TCRα, TCRβ, TCRδ*, and *TCRγ*. The aligned reads were assembled to extract CDR3 gene regions. To reduce false-positive CDR3 clonotype calls, the minimum number of supporting UMI was set as seven, more stringent than the manufacturer's recommendation of five. Shannon entropies for TCRα-, TCRβ-, TCRδ-, and TCRγ-chain sequences were calculated using the R package “vegan” from UMI counts for each patient.

### Metabolomics

Frozen tissue samples (*∼*10 mg) were immersed in methanol (500 μL) containing internal standards (20 μmol/L each of methionine sulfone and D-camphor-10-sulfonic acid) and homogenized using a Shake Master NEO (Bio Medical Science Co. Ltd.). Chloroform (500 μL) and Milli-Q water (200 μL) were added to the homogenate; it was thoroughly mixed and centrifuged at 4,600 × *g* for 15 minutes at 4°C. The upper aqueous fraction was centrifugally filtered through a Millipore 5-kDa cutoff filter (Human Metabolome Technologies) to remove proteins. The filtrate was dried using an evacuated centrifuge and dissolved in Milli-Q water (25 μL) containing 200 μmol/L of the reference compounds (3-aminopyrrolidine and trimesic acid) before capillary electrophoresis-mass spectrometry (CE-MS). CE-MS–based metabolomic profiling and data analysis were performed as described previously (16).

### Statistical Analyses

All analyses were performed using R version 4.0.1 (R Foundation for Statistical Computing). Mann–Whitney *U*, Kruskal–Wallis, and Fisher exact tests were used. Event-free survival (EFS) was defined as the time from surgery to induction failure using relapse or death resulting from any cause. EFS differences among groups were analyzed using the Kaplan–Meier method and log-rank test. Cox proportional hazards model was used for univariate and multivariate prognostic analysis using the R “survival” package. Statistical significance was set at *P* <0.05.

### Data Availability Statement

The raw data for whole-genome sequencing and RNA-seq are available under accession number hum0392 (JGAS000615) in the National Bioscience Database Center (NBDC). Access can be requested through the NBDC application system (https://humandbs.biosciencedbc.jp/en/data-use). Raw flow cytometry data are embargoed until April 2025 per the terms of our grant policy. Derived data supporting the findings of this study are available from the corresponding authors upon request. All other data presented within this article are available upon request to the corresponding authors.

## Results

### TIL Characteristics

To analyze immune cell counts in a prospective cohort of patients with NSCLC undergoing surgical resection (Supplementary Fig. S1), we employed an FCM panel of 26 markers to identify 30 unique immune cell types and functional subpopulations from 156 NSCLC and 157 NAT samples. The gating strategy is shown in Supplementary Fig. S2.

**FIGURE 1:**
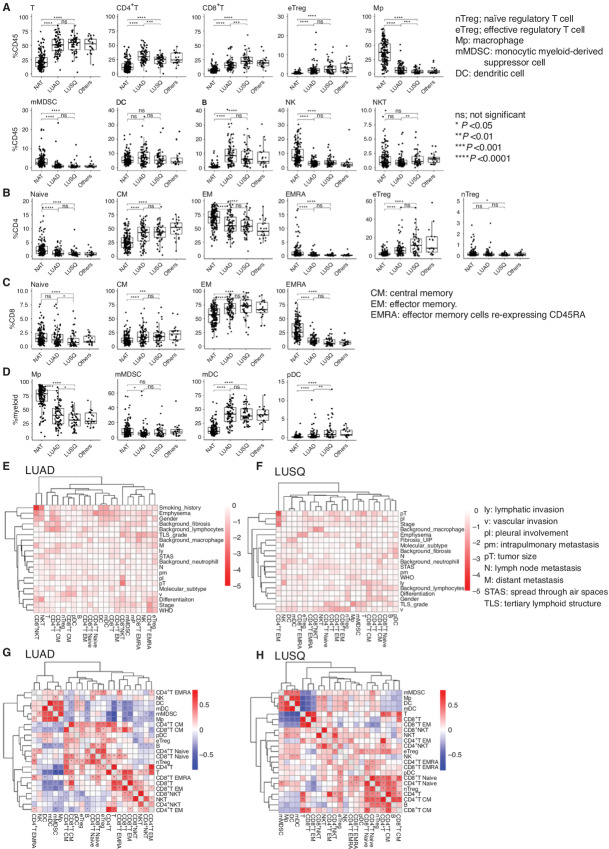
Immune cell compositions in LUAD and LUSQ; NAT (*n* = 157), LUAD (*n* = 85), LUSQ (*n* = 50), other types of lung cancer (*n* = 21). **A,** Percentages of immune cells per CD45^+^ cells (%CD45) in NSCLC and NATs. **B,** Percentages of CD4^+^ T-cell subsets per total CD4^+^ T cells for LUAD and LUSQ. **C,** Percentages of CD8^+^ T-cell subsets per total CD8^+^ T cells. **D,** Percentages of myeloid cell subsets per total myeloid cells. **E** and **F,** Relationships of immune cell types to clinicopathologic factors. Cells in the matrix represent log_10_ (Kruskal–Wallis *P* value) between %CD45 of immune cell compositions and clinicopathologic factors in LUAD (E) and LUSQ (F). Heat maps showing Spearman correlations between 23 immune cell types. Cell populations were clustered using hierarchical clustering in LUAD (**G**) and LUSQ (**H**).

We first compared TIL compositions between LUAD and LUSQ. Immune cell types per gram of tumor tissue (cell density) and percentage of immune cell type per CD45^+^ cells (%CD45) of CD8^+^ T and Natural killer T (NKT) cells were significantly higher in LUSQ than in LUAD (Fig. 1A). The %CD45 of CD4^+^ T cells and macrophages was higher in LUAD than in LUSQ. The %CD4 (percentage of CD4^+^ T-cell subset per total CD4^+^ T cells) of effector regulatory T cells (eTreg) was higher in LUSQ than in LUAD (Fig. 1B). The %CD8 (percentage of CD8^+^ T-cell subset per total CD8^+^ T cells) of naïve and effector memory (EM) cells reexpressing CD45RA (EMRA) CD8^+^ T cells was higher in LUAD than in LUSQ (Fig. 1C). The %myeloid (percentage of myeloid cell subset per total myeloid cells) of macrophages was higher in LUAD than in LUSQ, and that of plasmacytoid dendritic cells was higher in LUSQ than in LUAD (Fig. 1D). Detailed cell density analyses of TIL subsets are shown in Supplementary Fig. S3A–S3C.

### Correlations of TIL Composition with Clinicopathologic Factors

We then examined the relationships between immune cell types and histologic classifications (Supplementary Fig. S4 and S5). The %CD45 and %CD4 of naïve Tregs were higher in poorly differentiated LUSQ, and those of eTreg populations and CTLA-4^+^ eTregs were increased in poorly differentiated LUAD (Supplementary Fig. S4A, S4B, and S4E). The %CD8 of central memory (CM) CD8^+^ T (Supplementary Fig. S4C) and programmed cell death (PD)-1^+^ CM CD8^+^ T (Supplementary Fig. S4F) cells was higher in moderately and poorly differentiated types than in well-differentiated LUSQ. The %CD45 and %CD4 of eTregs were significantly higher in the solid type in LUAD (Supplementary Fig. S5A and S5B).

We next examined correlations between immune cell types and clinicopathologic factors. In LUAD, smoking history was strongly correlated with the %CD45 of CD8^+^ NKT cells (Fig. 1E). In LUSQ, pT was correlated with EM CD4^+^ T cells (Fig. 1F). Sex was associated with the cell density of T-cell and myeloid cell subsets in LUAD (Supplementary Fig. S6).

Positive correlations were observed between myeloid-type cells, including myeloid dendritic cells, monocytic myeloid-derived suppressor cells (mMDSCs), and macrophages. Positive correlations between T cells, including CD4^+^ and CD8^+^ T cells and their subsets, were also recognized in LUAD (Fig. 1G) and LUSQ (Fig. 1H). Conversely, the myeloid and T-cell types showed mutually exclusive relationships.

### Immunological Classification Using TIL Profiling

**FIGURE 2:**
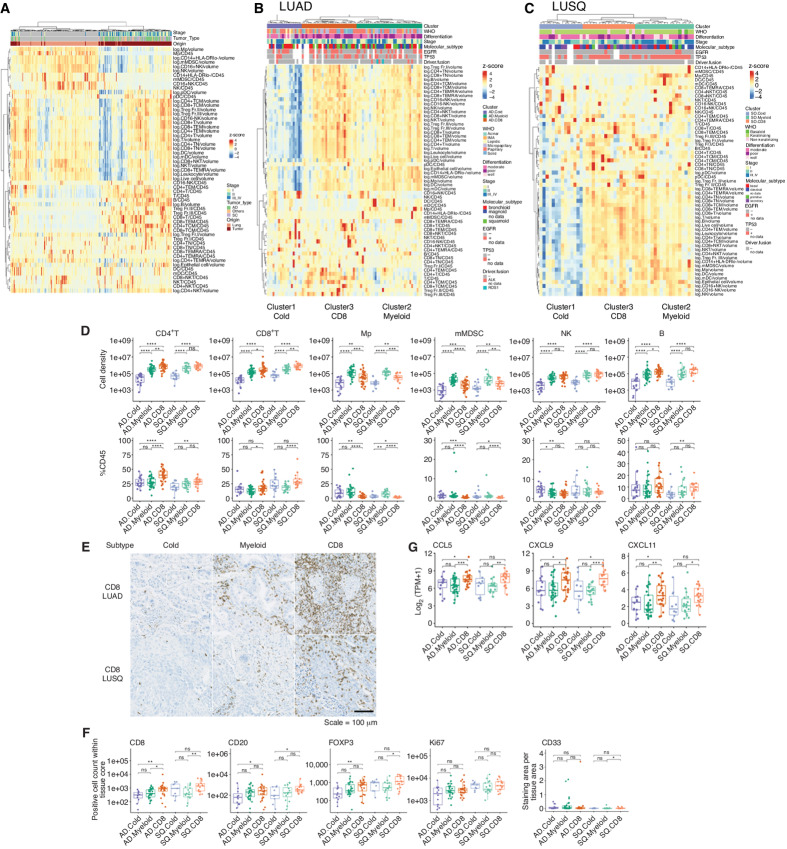
Immune subtypes based on TIL profiling using FCM; NAT (*n* = 157), LUAD [85; Cold (*n* = 19), Myeloid (*n* = 36), CD8 (*n* = 30)], LUSQ [50; Cold (*n* = 14), Myeloid (*n* = 19), CD8 (*n* = 17)]. **A,** Heat map of cell densities and %CD45 of immune cells in NSCLC tumors and NATs. Each column represents a patient; data were normalized by Z-scores to indicate relative compositions of immune cells. Cell density is presented as the log of each cell type. %CD45 is presented as each immune cell type/CD45. Heat maps of cell density and %CD45 of TILs in LUAD (**B**) and LUSQ (**C**) tissues. **D,** Cell densities and %CD45 of immune cell types in LUAD and LUSQ immune subtypes. Top, Cell density indicates immune cells. Bottom, %CD45 indicates immune cells. AD.Cold, AD.Myeloid, and AD.CD8 are LUAD subtypes Cold, Myeloid, and CD8, respectively. SQ.Cold, SQ.Myeloid, and SQ.CD8 are LUSQ subtypes Cold, Myeloid, and CD8, respectively. **E,** IHC staining for CD8 in a representative case from immune subtypes. For CD8, FOXP3, CD20, and Ki67, the positivity of each marker was given as the number of total positive cell number within each tissue core. For CD33, the ratio of CD33-positive staining area per tissue area was calculated. **F,** Immune cell counts by IHC staining in lung cancer tissues. **G,** CD8^+^ T cell–attractant chemokine gene expression in immune subtypes.

Unsupervised clustering using FCM data was performed to assess differences in immune cell composition between tumors and NATs. Clustering with cell density and %CD45 of immune cell types revealed two major clades (Fig. 2A): one (right branch of dendrogram) comprising almost all NSCLC tissues, and the other (left branch) comprising almost all NATs.

We then examined TME immunological characteristics. Following unsupervised clustering, LUAD and LUSQ were clustered into three distinct TIL profiles (Fig. 2B and C). In Cluster 1 of both LUAD and LUSQ, the numbers of infiltrating immune cells were notably lower than those in the other two clusters. Clusters 2 and 3 showed many infiltrating leukocytes in tumor tissues. In Cluster 2, the %CD45 of myeloid cells, including macrophages, CD14^+^ monocytes, and mMDSCs, which are immune suppressive (17), was increased, whereas the %CD45 of T cells, including CD8^+^ and EM CD8^+^ T, was decreased compared with that in Cluster 3. In contrast, in Cluster 3, the %CD45 of the above myeloid lineage cells was reduced and that of T cells (both CD4^+^ and CD8^+^ and their EM and CM subsets), which are considered effector cells (18), increased compared with that in Cluster 2. We termed Cluster 1 as the cold subtype (Cold), Cluster 2 as the myeloid cell–dominant subtype (Myeloid), and Cluster 3 as the CD8^+^ T cell–dominant subtype (CD8).

Next, we validated the cell density and percentage of each immune cell in immune subtypes using FCM data. Cell densities and %CD45 of T, CD4^+^ T, CD8^+^ T, and B cells were higher in subtype CD8 than those in the Cold and Myeloid subtypes in both LUAD and LUSQ (Fig. 2D; Supplementary Fig. S7A). The %CD4, %CD8, and %myeloid in immune subtypes were presented in Supplementary Fig. S7B–S7D. In contrast, cell densities and %CD45 of myeloid cells, including macrophages and mMDSCs, were higher in the Myeloid subtype than those in the Cold and CD8 subtypes (Fig. 2D). The increased number of CD8^+^ T and B cells in the CD8 subtype was confirmed using IHC of tissue microarrays (Fig. 2E and F; Supplementary Fig. S8A and S8B). The ratio of CD33-positive staining area per tissue area was calculated because of the difficulty to recognize one cell of myeloid cell lineage by Halo system. The ratio was considerably lower than the number of CD8^+^ T and CD20^+^ cells within each tissue core, and no significant difference was detected among immune cell types (Fig. 2F; Supplementary Fig. S9A and S9B). Detailed analyses of CD4^+^ T, CD8^+^ T, and myeloid cells using FCM in immune subtypes are presented in Supplementary Fig. S7B–S7D. In addition, the expression of CD8^+^ T cell–trafficking chemokine genes (*CCL5*, *CXCL9*, and *CXCL11*) was significantly higher in subtype CD8 than that in the Cold and/or Myeloid subtypes in LUAD and LUSQ, which is one reason for the CD8^+^ T cell–rich TME in subtype CD8 (Fig. 2G).

### Correlating Immune Subtypes with Patient Outcomes

**FIGURE 3:**
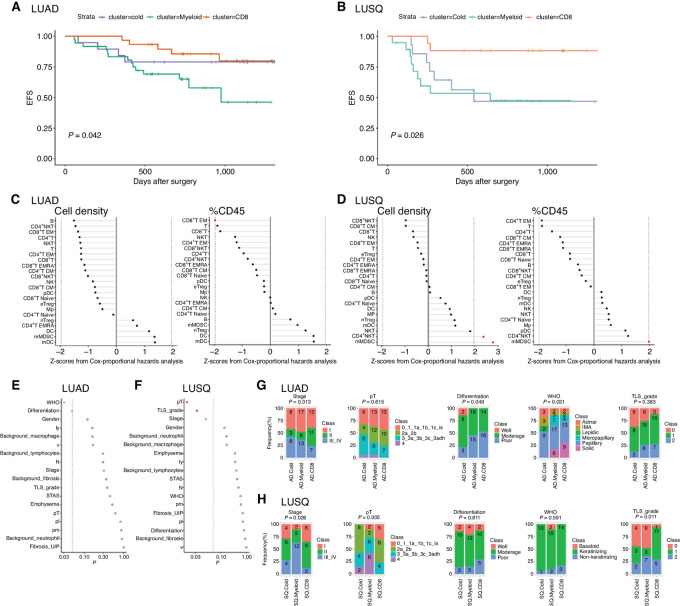
Patient outcomes in immune subtypes. **A** and **B,** Kaplan–Meier EFS curves for subtypes of LUAD (A) and LUSQ (B). *P* values were calculated by multivariate Cox regression. **C** and **D,** Relationships of cell density (left) with %CD45 (right) for each immune cell type infiltrated in tumors to patient prognosis in LUAD (C) and LUSQ (D). Z-scores from Cox proportional hazards analysis are plotted; in the plots, red dots indicate *P* ≤ 0.05. **E** and **F,** Differences in histopathologic findings for immune subtypes of LUAD (E) and LUS (F). The *P* values determined using Fisher exact test w are plotted; in the plots, red circles indicate *P* ≤ 0.05 and filled red circles indicate Holm-adjusted *P* ≤ 0.2. **G** and **H,** Frequencies of each classification in immune subtypes in LUAD (G) and LUSQ (H). Numbers of patients are indicated in the bar segments.

To demonstrate the clinical significance of our subtyping, we examined patient outcomes (EFS) as a function of the immune subtype. The EFS of subtype CD8 was significantly longer than that of subtype Myeloid in LUAD (Fig. 3A) and was longer than that of Cold and Myeloid subtypes in LUSQ (Fig. 3B). However, no significant differences were observed in EFS between Cold and Myeloid immune subtypes or between Myeloid and CD8 immune subtypes in LUAD (Fig. 3A).

We then examined immune cell types associated with patient outcomes. The %CD45 of EM CD8^+^ T cells was significantly associated with a better prognosis in LUAD (Fig. 3C). Both mMDSCs and CD4^+^ NKT cells were associated with a poorer prognosis in LUSQ (Fig. 3D).

To demonstrate the clinical usefulness of TIL-based classification of immune TME, we examined the significance of various factors, including clinicopathologic variables, molecular subtyping, and TIL subtyping, on the prediction of patient outcomes. Univariate analysis showed that pT, ly, v, pl, N, STAS, pathologic stages, TIL subtyping (CD8 vs. Myeloid), and tumor-positive surgical margin were significant prognostic factors of EFS in LUAD (Table 1). Smoking index, pl, N, pathologic stages, TIL clustering (CD8 vs. Myeloid and CD8 vs. Cold), and tumor-positive surgical margin were prognostic factors of EFS in LUSQ. Multivariate analysis with significant factors by univariate analysis revealed that pT, ly, N, and TIL subtyping (CD8 vs. Myeloid) were independent prognostic factors for EFS in LUAD. There were no independent prognostic factors for EFS in LUSQ.

**TABLE 1:** Univariate and multivariate analyses of prognostic factors in LUAD and LUSQ

		Univariate				Multivariate			
		EFS: exp(−coef)	EFS:lower 0.95	EFS:upper 0.95	EFS:*P*	EFS: exp(−coef)	EFS:lower 0.95	EFS:upper 0.95	EFS:*P*
**LUAD**									
Age	<65 vs. ≥65	1.500	0.590	3.811	0.37984				
Gender	female vs. male	1.082	0.477	2.453	0.85100				
Smoking_index	<800 vs. ≥800	1.681	0.690	4.097	0.27088				
Tumor_size	<30 vs. ≥30	9.127	2.138	38.958	0.00007	5.398	1.037	28.085	0.04512
Differentiation	well-moderate vs. poor	1.062	0.440	2.565	0.89291				
ly	ly0 vs. ly1	2.567	1.120	5.883	0.02557	0.066	0.006	0.743	0.02779
v	v0 vs. v1–3	3.916	1.163	13.193	0.01008	2.658	0.352	20.092	0.34350
pl	pl0 vs. pl1–3	3.399	1.469	7.864	0.00374	1.650	0.476	5.725	0.43008
pm	pm0 vs. pm1–2	1.446	0.534	3.917	0.48364				
N	0 vs. 1–3	5.591	2.294	13.626	0.00006	13.954	1.179	165.124	0.03655
STAS	0 vs. 1	5.074	1.715	15.016	0.00076	3.161	0.662	15.090	0.14902
Stage	I–II vs. III–IV	5.957	2.443	14.527	0.00003	0.762	0.134	4.336	0.75886
Molecular_subtype	bronchioid+squamoid vs. magnoid	2.061	0.851	4.986	0.12230				
TIL_CD8_Myeloid	CD8 vs. Myeloid	3.406	1.207	9.613	0.01309	15.268	1.902	122.563	0.01032
TIL_CD8_Cold	CD8 vs. Cold	1.369	0.367	5.105	0.64278				
Surgical_margin	negative vs. positive	4.502	1.755	11.550	0.00610	1.697	0.434	6.633	0.44700
**LUSQ**									
Age	<65 vs. ≥65	0.679	0.255	1.810	0.44980				
Gender	female vs. male	0.982	0.284	3.395	0.97671				
Smoking_index	<800 vs. ≥800	5.414	0.719	40.792	0.03414	2.855	0.306	26.659	0.35740
Tumor_size	<30 vs. ≥30	1.171	0.385	3.559	0.77797				
Differentiation	well-moderate vs. poor	0.864	0.250	2.989	0.81506				
ly	ly0 vs. ly1	1.640	0.647	4.160	0.30407				
v	v0 vs. v1–3	75176989.892	0.000	Inf	0.08536				
pl	pl0 vs. pl1–3	3.986	1.302	12.202	0.00756	2.708	0.362	20.277	0.33215
pm	pm0 vs. pm1–2	3.211	0.727	14.189	0.18224				
N	0 vs. 1–3	2.709	1.068	6.872	0.03629	2.870	0.382	21.563	0.30544
STAS	0 vs. 1	1.008	0.375	2.709	0.98690				
Stage	I–II vs. III–IV	4.957	1.849	13.288	0.00095	1.024	0.071	14.857	0.98602
Molecular_subtype	classical+secretory vs. primitive+basal	1.468	0.492	4.384	0.50044				
TIL_CD8_Myeloid	CD8 vs. Myeloid	6.051	1.302	28.122	0.00785	3.629	0.381	34.601	0.26264
TIL_CD8_Cold	CD8 vs. Cold	5.493	1.135	26.575	0.01795				
Surgical_margin	negative vs. positive	6.777	2.515	18.257	0.00062	3.035	0.477	19.293	0.23944

To clarify the histopathologic characteristics of the respective immune subtypes, we examined them as a function of the subtype. WHO classifications and histologic differentiation grades were significantly different in LUAD immune subtypes (Fig. 3E). In contrast, for LUSQ subtypes, pT, TLS grade, and stage were different (Fig. 3F). Specifically, in LUAD subtype Cold, the frequency of poorly differentiated cases was lower than those in the other two subtypes, and micropapillary and solid types in the WHO classification were not recognized (Fig. 3G). In LUSQ subtype Myeloid, cases with stage III/IV and pT class4 were more common than in the other two subtypes. The TLS grade was higher in subtype CD8 than in the other subtypes in LUSQ (Fig. 3H). Detailed analyses of histopathologic factors in the respective immune subtypes are presented in Supplementary Fig. S10A and S10B.

### Immune Subtypes are Related to TME Immune Activation Status

**FIGURE 4:**
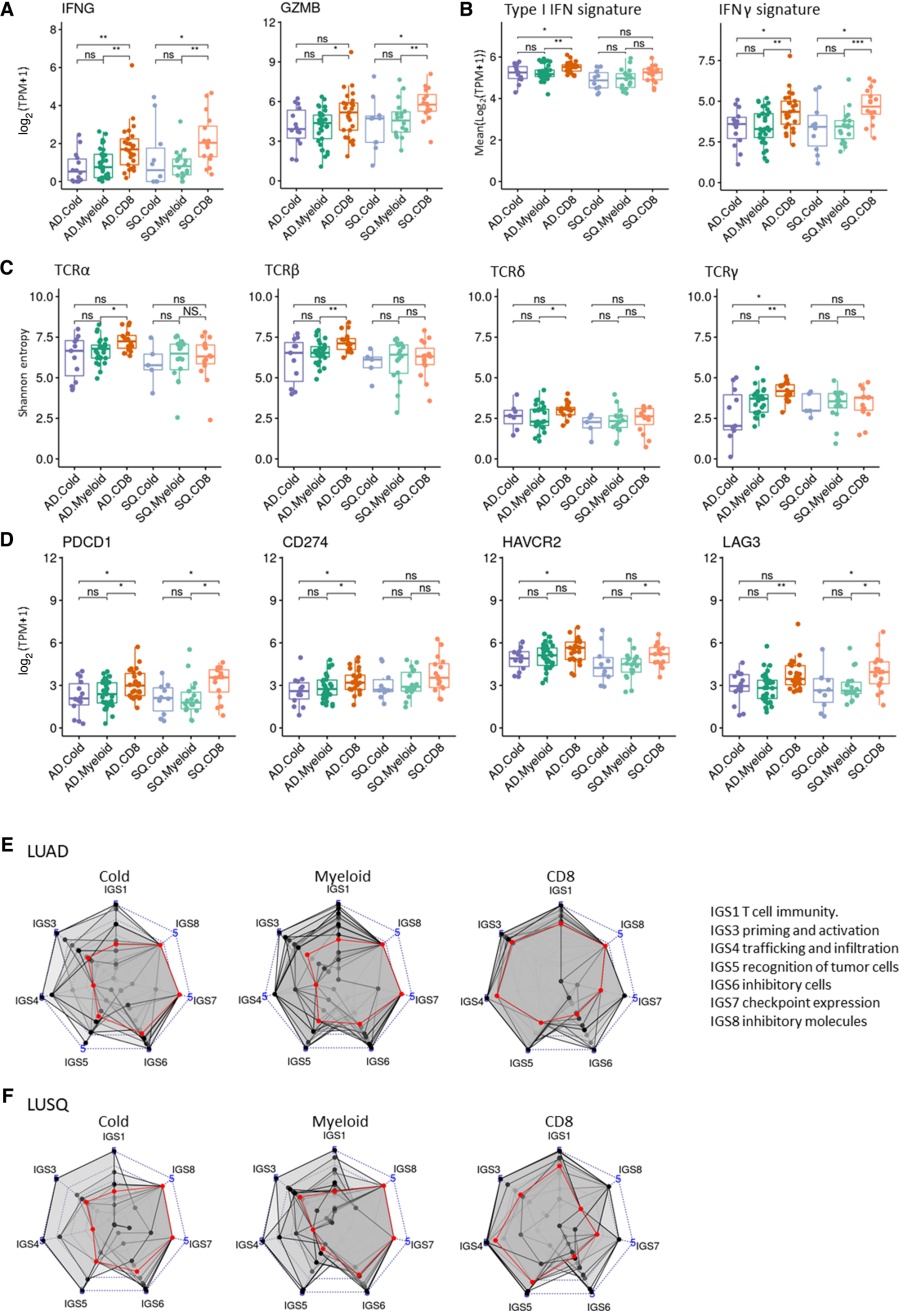
Immunologic characteristics of immune subtypes based on TIL profiling. Numbers of cases analyzed using RNA-seq were LUAD (*n* = 72; Cold, *n* = 14; Myeloid, *n* = 33; CD8, *n* = 25) and LUSQ (*n* = 44; Cold, *n* = 10; Myeloid, *n* = 18; CD8, *n* = 16). **A,** Expression of IFNγ and granzyme B as a function of immune subtype. **B,** Scores of type I IFN and IFNγ signatures as a function of immune subtype. Expression levels and scores were obtained from RNA-seq. **C,** Boxplots showing Shannon entropies of canonical TCR repertoires (TCRα, TCRβ, TCRδ, and TCRγ) as a function of immune subtype. **D,** Expression of immune checkpoint molecules as a function of immune subtype. *PDCD1*, *CD274*, *HAVCR2*, and *LAG3* expression levels are obtained from RNA-seq. **E** and **F,** Radar plots showing immunograms of immune subtypes; axes were generated with the IGS. Median IGS is plotted in red. LUAD (E). LUSQ (F).

We next examined the TME immune activation status in the context of the immune subtype. RNA-seq showed that in both LUAD and LUSQ, the expression of IFNγ and granzyme B genes, as well as scores of type I IFN (www.ebi.ac.uk/QuickGO/term/GO:0060337) and IFNγ signatures (19) were higher in subtype CD8 than in the other two subtypes (Fig. 4A and B). TCR repertoire diversity, including TCRα, TCRβ, TCRδ, and TCRγ, was highest in subtype CD8 in LUAD (Fig. 4C). Immunoreactivity was induced to a greater extent in subtype CD8 than in the other two subtypes.

Next, we examined the expression of immune checkpoint molecules in the context of immune subtypes. *PDCD1* (PD-1), *CD274* (PD-L1), *HAVCR2* (TIM3), and *LAG3* expression was elevated in subtype CD8 compared with that in the other two subtypes in LUAD and LUSQ (Fig. 4D). The naïve and CM CD8^+^ T-cell subsets in LUAD subtype CD8 showed a higher percentage of PD-1^+^ and PD-L1^+^ fractions than did subtype Cold (Supplementary Fig. S11A and S11B), suggesting the subtype CD8 has an inflamed TME.

Metabolomic analysis showed that the level of tryptophan, which induces immunostimulatory cytokines and activates T cells (20), was higher in subtype CD8 than that in subtype Myeloid in LUAD (Supplementary Fig. S11). In both LUAD and LUSQ, the level of the immunosuppressive metabolite kynurenine (21) did not change between subtypes Myeloid and CD8, indicating that it is likely not involved in the immunosuppressive TME in subtype Myeloid (Supplementary Fig. S12). No significant difference was observed in molecular subtypes (11, 12) between immune subtypes in LUAD and LUSQ (Supplementary Fig. S13).

### Cancer-immunity Cycle Disruptions

Immunograms were constructed to understand which processes in the cancer-immunity cycle were disturbed in inducing immune reactions in NSCLC tissues. Immunograms were examined in respective immune subtypes (ref. 13; Fig. 4E and F). In LUAD and LUSQ subtypes Cold and Myeloid, the early steps, including T-cell immunity, priming and activation, T-cell trafficking and infiltration, and tumor cell recognition, were broadly disrupted. Although an immune reaction was induced in subtype CD8 (Fig. 4A and B), the recognition of tumor cells, inhibitory cells, and immune checkpoint expression in subtype CD8 of LUAD, and the inhibitory cells and inhibitory molecules in subtype CD8 of LUSQ remained weak points on the immunogram.

### Specific Gene Expression Pathways in Immune Subtypes

**FIGURE 5:**
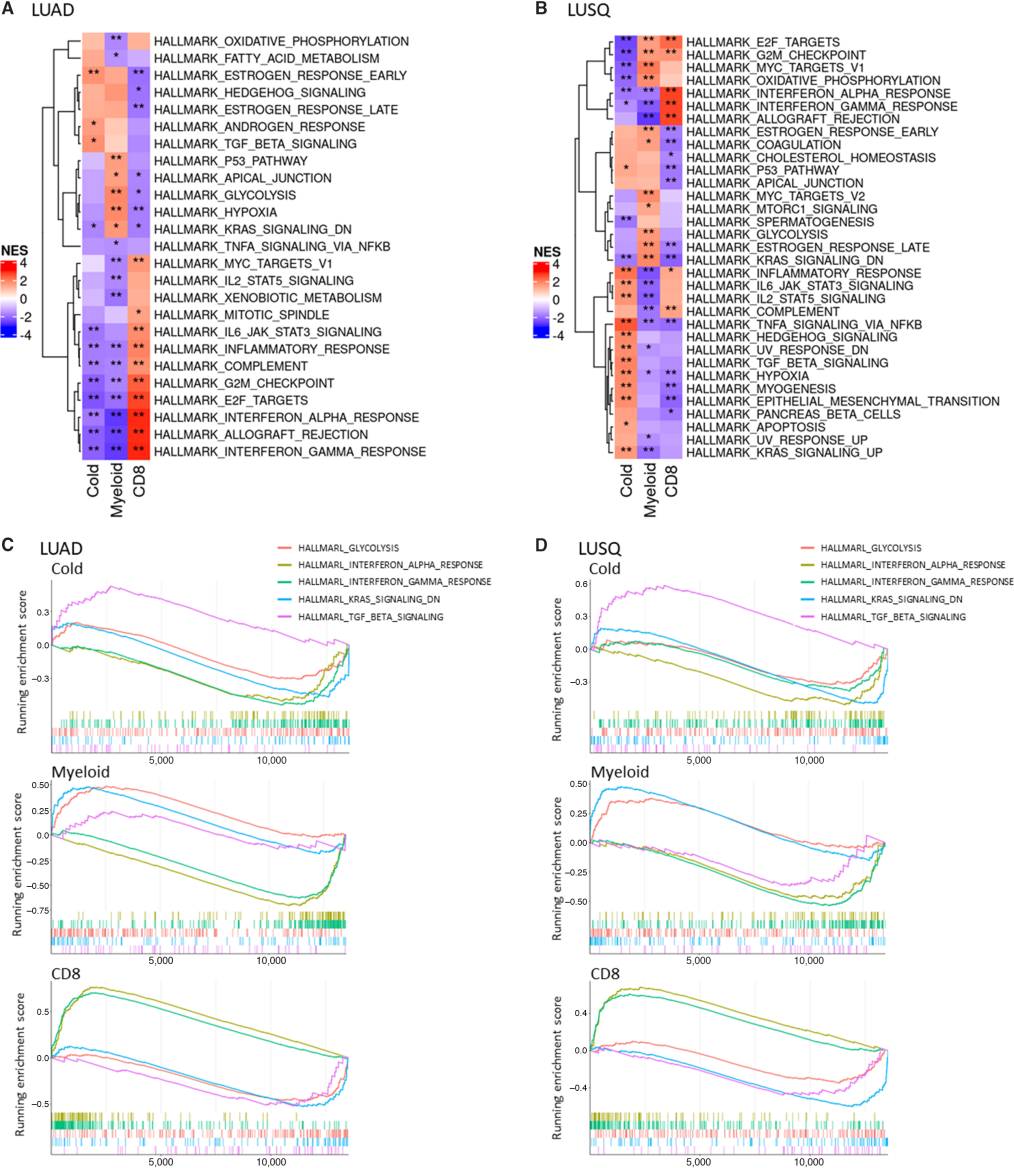
Activated and suppressed pathways identified by GSEA using the hallmark gene set in immune subtypes. **A** and **B,** Heat maps representing top scored pathways enriched in genes with expression increased and decreased in common in LUAD (A) and LUSQ (B). **C** and **D,** Top signatures of pathways with increased expression are shown in red; those for genes with decreased expression are shown in blue. Running enrichment scores for glycolysis, IFNα, IFNγ, K-*ras* signaling, and TGFβ signaling signatures as commonly activated pathways in immune subtypes of LUAD (C) and LUSQ (D).

GSEA using the hallmark gene set clarified that expression of components of the TGFβ pathway was significantly upregulated in subtype Cold, and the signatures of glycolysis and K-*ras* signaling were upregulated in subtype Myeloid, compared with those in the other two subtypes common to LUAD and LUSQ (Fig. 5A–D). In LUAD and LUSQ subtype CD8, immune-related signatures, including IFNα response, IFNγ response, and allograft rejection, were upregulated over those in the other two subtypes, consistent with the aforementioned findings of enhanced TIL infiltration and immune activation status (Figs. 2D, 4A, and B). Conversely, the first two of these pathways, IFNα and IFNγ responses, were downregulated in LUAD and LUSQ subtypes Cold and Myeloid (Fig. 5A–D).

GSEA using the GO set revealed that signatures of epidermis-related pathways, including epidermis development, skin development, and keratinocyte differentiation, were upregulated in LUAD and LUSQ subtype Myeloid compared with those in the other two subtypes (Supplementary Fig. S14A–S14D). The upregulation of immune-related pathways was confirmed in subtype CD8 using the GO set. Circulation system-related pathways, including blood vessel morphogenesis, cardiac chamber morphogenesis, and cardiac muscle tissue morphogenesis, were upregulated in subtype Cold.

To understand the molecular mechanisms underlying the poorer prognosis of subtype Myeloid, we examined the relationship between the signatures identified in subtype Myeloid of LUAD and patient prognosis using transcriptome data from The Cancer Genome Atlas NSCLC. The upregulated signatures included glycolysis, hypoxia, and apical junctions by the hallmark gene set (Supplementary Fig. S15A). The epidermis-related pathways, including keratinization, skin development, and epidermis development, were identified to be upregulated by the GO set (Supplementary Fig. S15B). These upregulated signatures were significantly associated with a poor prognosis in LUAD and represent possible targets for improving the immunosuppressive TME.

### Characteristic Subtype Genomic Alterations

**FIGURE 6:**
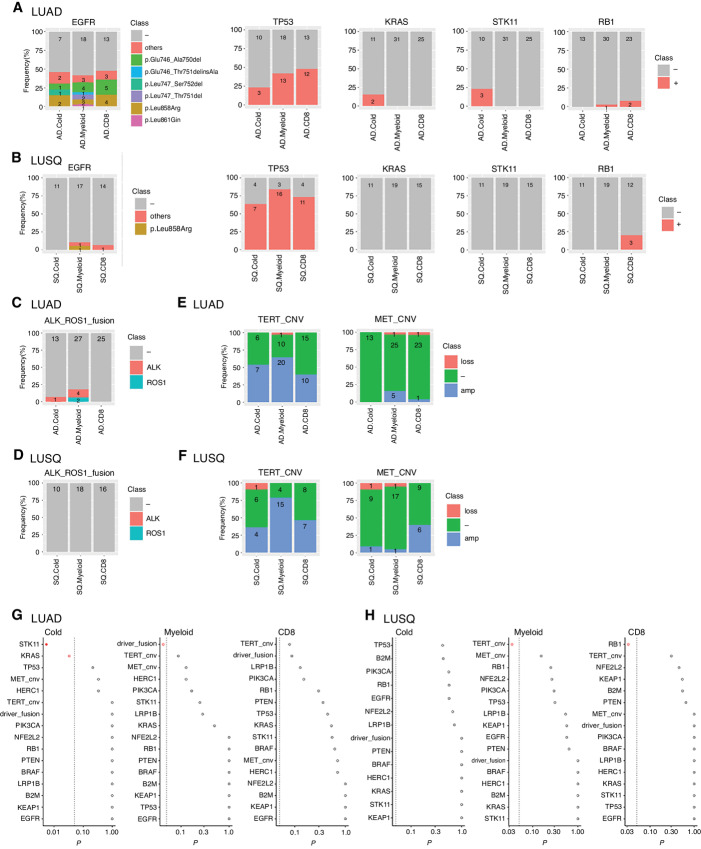
Characteristics of genomic alterations as a function of immune subtype. Numbers of cases analyzed using WES were LUAD (*n* = 69; Cold, *n* = 13; Myeloid, *n* = 31; CD8, *n* = 25), and LUSQ (*n* = 45; Cold, *n* = 11; Myeloid, *n* = 19; CD8, *n* = 15). Number of cases analyzed using RNA-seq for fusion genes are the same as described in Fig. 4. **A–F,** Characteristics of gene alterations. Number of patients are indicated in each column. Mutations of *EGFR, TP53, KRAS, STK11,* and RB1 in LUAD (**A**) and LUSQ (**B**) were analyzed using WES. *ALK* and *ROS1* fusion genes in LUAD (**C**) and LUSQ (**D**) were analyzed with RNA-seq. Amplification or loss of *MET* and *TERT* in LUAD (**E**) and LUSQ (**F**) were analyzed using WES. Differences in gene alterations for immune subtypes in LUAD (**G**) and LUSQ (**H**) plotted as *P* values determined using Fisher exact test w. In the dot plots, red circles indicate *P* ≤ 0.05 and the filled red circle indicates Holm-adjusted *P* ≤ 0.2.

Tumor genomic mutations affect immunoreactivity in the TME (22); therefore, we examined whether our three immune subtypes were associated with specific genomic alterations using WES and RNA-seq data. First, the tumor mutation burden was not significantly different across subtypes in LUAD and LUSQ (Supplementary Fig. S16). The frequency of *EGFR* mutations was much higher in LUAD (46.5%) than in LUSQ (6.7%), and the frequency of *TP53* mutations was higher in LUSQ (75.6%) than in LUAD (39.4%; Fig. 6A and B). Cases with driver fusion genes containing *ALK* (five cases; 6.9%) and *ROS1* (two cases; 2.8%) were present in LUAD but not in LUSQ (Fig. 6C and D). Finally, gene copy-number variation (CNV) of *MET* and *TERT* was recognized in both LUAD and LUSQ (Fig. 6E and F). Furthermore, we analyzed the mutation of *ERBB2, NRAS, HRAS, FGFR1, FGFR2,* and *RIT1*, which were found at a certain rate as reported previously (23, 24). However, because the number of cases with these gene mutations detected in this cohort was small (*FGFR2* mutation in Cold subtype of LUAD: *n* = 1, NRAS mutation in CD8 subtype of LUAD: *n* = 1, HRAS mutation in CD8 subtype of LUSQ: *n* = 1), we could not detect a statistically significant difference in the frequency of these gene mutations among immune subtypes.

With respect to subtype specificity, the frequencies of *EGFR* and *TP53* mutations were not different among the three immune subtypes (Fig. 6G and H). *KRAS* and *STK11* mutations were enriched in subtype Cold. *ALK* or *ROS1* fusion genes were enriched in the subtype Myeloid compared with those in the other two subtypes in LUAD (Fig. 6G). In LUSQ, the frequency of *TERT* CNV was higher in subtype Myeloid, and *RB1* mutation was enriched in subtype CD8 compared with that in the other two subtypes (Fig. 6H). The degree of *TERT* CNV was significantly correlated with the frequency of mMDSCs and macrophages, but not with T and CD8^+^ T cells (Supplementary Fig. S17). These genomic alterations may orchestrate immune TME construction in the respective immune subtypes.

## Discussion

We used multicolor FCM to assess TILs in NSCLC surgical specimens. TIL characteristics in lung cancer tissues were consistent with those reported previously (25). Unsupervised clustering of the TIL profile allowed us to classify both LUAD and LUSQ into three immune subtypes that correlated with patient outcomes. We examined their clinicopathologic, immunobiological, and genomic characteristics, identifying subtype-specific molecular pathways and genomic alterations that should play important roles in constructing subtype-specific immune TMEs.

Patient outcomes for subtype Cold were not statistically different from those for CD8 in LUAD. Such “cold” tumors have a poor prognosis for multiple cancers (4); however, this phenomenon appears to be cancer type specific. In several types, such as uterine cervical and renal cancers, no survival difference was observed between the desert and inflamed tumors (26). The frequency of poorly differentiated types was significantly lower, and the micropapillary and solid types, which were related to poor prognosis (27), were not included in the Cold subtype of LUAD, which could explain the fact that subtype Cold showed not bad prognosis in this study.

Regarding the association of immune cell types with patient outcomes, mMDSCs and CD4^+^ NKT cells were significantly related to a poor prognosis in LUSQ. NKT cells skew the immune response toward both inflammation and tolerance by secreting various cytokines (28). Although the role of CD4^+^ NKT cells is not established in lung cancer, these cells show no cytotoxic activity against tumor cells (29). Moreover, the potential of the CD4^+^CD8^−^ NKT cell subset to suppress the immune response by secreting IL4 and IL10 has been demonstrated in various mouse models (28). Therefore, CD4^+^ NKT cells may be associated with a poor prognosis in LUSQ.

On the basis of transcriptomic analyses, TME of NSCLC is categorized into the immunity-high, immunity-medium, and immunity-low subtypes (30–32), and proteogenomic analysis of LUAD identified three major clusters (hot, cold-tumor enriched, and cold-NAT enriched; ref. 33). Pan-cancer immunogenic analyses based on transcriptomics and immunophenoscore data have identified four (conserved across 20 cancer types), and six (conserved across 33 cancer types) TME subtypes, respectively (26, 34, 35). However, the distinction between myeloid cell–dominant and CD8^+^ T cell–dominant types in the TIL-enriched TME is limited by the accuracy of transcriptome and proteogenomic TIL profiling, which could be addressed using FCM immune cell profiling. Considering stromal components, including cancer-associated fibroblasts (CAF) and vascular endothelial cells, it is essential to understand the immune TME as a whole (26). Therefore, we plan to include stromal components in our subtyping platform in a future study.

Why did individual NSCLC tissues have different immunological TMEs? GSEA showed distinct pathways in immune types; the immunoreactivity-related pathways were mainly activated in the subtype CD8 in both LUAD and LUSQ. GSEA using the hallmark gene set, revealed that the TGFβ pathway was activated in the Cold subtype. TGFβ induces the activation of tumor-supporting CAFs, creating a physiologic barrier around the TME that hampers immune cell influx (36). In the Myeloid subtype, glycolysis and K-*ras* signaling pathways were upregulated compared with those in the other two subtypes. MDSC proliferation is supported by the glycolytic metabolite phosphoenolpyruvate in the TME, and MDSCs exhibit higher glycolytic activity (37). In addition, increased glycolysis is associated with MDSC promotion through AMPK-ULK1 and autophagy in breast cancer (38). In terms of K-*ras* signaling, oncogenic KRAS represses IRF2 expression, leading to high CXCL3 expression, which recruits MDSCs in the TME (39). The GSEA using the GO set showed that epithelial differentiation pathways, as well as multiple laminin and keratin genes, were activated in the Myeloid subtype. CAF-mediated upregulation of laminin-γ2 constructs hermetic shields surrounding tumors, impeding T-cell penetration into the tumor nests (40). These Myeloid subtype–specific pathways are potential targets for developing immunotherapies or strategies to enhance ICB efficacy.

We propose a novel immunologic classification of NSCLC based on TIL profiling, which may be useful for determining patient prognosis. Multiple TILs were analyzed by FCM using surgically resected specimens. Although biopsies are a realistic option in clinical settings to evaluate predictive biomarkers, FCM requires several materials, limiting its application to tumor biopsies. Therefore, it is important to develop prognostic predictors employing IHC staining of fewer immune cell subsets in biopsy samples to apply this immune classification in clinical settings. However, recognizing one cell of myeloid cell lineage using the digital image analysis is difficult owing to the diverse morphology of the myeloid cells. Moreover, our results showed a considerably lower ratio of CD33 staining area per tissue area than the number of CD8^+^ T and CD20^+^ cells within each tissue core. In addition, owing to the heterogeneity of myeloid cells, the combination of multiple cell surface markers is necessary to define various types of myeloid cells, such as MDSC (CD11b^+^CD33^+^ HLA-DR^low^). Therefore, it is essential to develop the optimal detection method for myeloid lineage cells based on IHC.

Genetic abnormalities of cancer cells shape the immune TME (22). Alterations in *FAK*, *PTEN*, *EGFR*, *WNT-β-catenin*, and *RHOA* have been associated with immunosuppressive TMEs (22, 41). We found that *KRAS*, *STK11*, *TERT*, *RB1* gene abnormalities, and *ALK* and *ROS1* fusions were associated with respective immune subtypes. Recently, it has been reported that *STK11* alteration inactivates the stimulator of interferon genes and downregulates T cell–recruiting chemokines such as CXCL10 (42). *TERT* activates a subclass of endogenous retrovirus (ERV), and ERV/interferon signaling stimulates the chemokine secretion, which attracts a suppressive T-cell population and MDSCs (43). However, in-depth studies are required to investigate the underlying mechanisms associated with the immune TMEs induced by these gene alterations.

Our immune subtypes are characterized by specific signaling pathways and gene abnormalities. The manipulation of specific pathways and responsible genes selected on the basis of these immune subtypes may allow reversing immunosuppressive TMEs to become antitumor ones, enhancing the antitumor immunity of ICBs, and leading to personalized, immune TME-based immunotherapies.

## Supplementary Material

Fig. S1 The number of patients and analyses in LUAD and LUSQ. The immunohistochemistry of tissue microarray, whole exome sequencing, TCR repertoire analysis, and metabolome analysis were performed in the cases FCM was conducted.

Fig. S2 Representative polychromatic dot plots demonstrating the gating strategy employed to identify the immune cell profile of TILs in NSCLC. Starting at the top left, the initial gate was to obtain live cells; next, leukocytes (CD45+) and epithelial cells (CD326+) were gated; finally, CD3+ cells and CD3- cells were separated. For CD3+ cells, a size gate was applied to identify NKT cells (CD3+ CD56+), CD3+CD4+ and CD3+CD8+ T cells. T cell subsets are displayed, including naïve (CD45RA+CD197+), central memory (CM: CD45RA-CD197+), effector memory (EM: CD45RA-CD197-), and effector memory cells re-expressing CD45RA (EMRA: CD45RA+CD197-). FOXP3-expressing CD4+ T cells were gated on CD4+ T cells: naïve Treg (Fr. I: CD45RA+FOXP3lo), effector Treg (Fr. II: CD45RA-FOXP3hi), and non-Treg (Fr. III: CD45RA-FOXP3lo). For CD3- cells, a size gate was applied to identify B (CD3-CD19+) and NK cells (CD3-CD56+). The myeloid cell lineage was also gated on CD3- cells: conventional DC (HLA-DR+CD11c+), plasmacytoid DC (HLA-DR+CD11c-CD123+), macrophage (CD68+SSChi) and monocytic MDSC (HLA-DRlo CD14+CD11b+CD33+). Ki-67-expressing cells were gated in CD8+NKT, CD4+NKT, CD4+T (NT, CM, EM, EMRA), CD8+T (NT, CM, EM, EMRA), Treg, B, NK, and mMDSC. PD-1-expressing cells were gated in CD4+T (naive, CM, EM, EMRA), CD8+T (naive, CM, EM, EMRA) and Treg cells. PD-L1-expressing cells were gated in CD8+T (NT, CM, EM, EMRA), and mMDSC. CTLA-4-expressing cells were gated in Treg cells.

Fig. S3 Number of each immune cell type, CD4+ T cell subset, and CD8 T+ cell subset by FCM from lung cancer and NATs. (a) Cell density of each immune cell type. Data are presented as the number of indicated immune cells per gram of NAT, LUAD, LUSQ, and other types of lung cancer tissues. (b, c) Density of CD4+ T cell subset (b) and CD8+ T cell subset (c). ns; not significant. * p<0.05. **P<0.01. ***P<0.001. ****P<0.0001.

Fig. S4 Relation of immune cell types with histological grade. LUAD (n=77) (well differentiated; n=2, moderately differentiated; n=41, poorly differentiated; n=34) and LUSQ (n=50) (well differentiated; n=8, moderately differentiated; n=32, poorly differentiated; n=10). (a–d) %CD45 of immune cell type (a), %CD4 of CD4+ T cell subset (b), %CD8 of CD8+ T cell subset (c), and %myeloid of myeloid cell type (d) are presented in well, moderately, and poorly differentiated LUAD and LUSQ. (e) %CD4 of CTLA-4+ eTregs in LUAD and LUSQ. (f) %CD8 of PD-1+ and PD-L1+ CM CD8+ T cells in LUAD and LUSQ. ns; not significant. * p<0.05. **P<0.01.

Fig. S5 Relation of immune cell types with WHO classification. LUAD (n=82) (lepidic; n=2, acinar; n=7, papillary; n=39, micropapillary; n=11, solid; n=15, IMA; n=8) and LUSQ (n=50) (non-keratinizing; n=6, keratinizing; n=42, basaloid; n=2). (a–d) %CD45 of immune cell type (a), %CD4 of CD4+ T cell subset (b), %CD8 of CD8+ T cell subset (c), and %myeloid of myeloid cell type (d) are presented following the WHO classification of LUAD and LUSQ. ns; not significant. * p<0.05. **P<0.01. ***P<0.001.

Fig. S6 Relationship between the number of immune cell types and clinicopathological factors. Cells in the matrix represent the 1-Pearson correlation coefficient between cell density of the indicated immune cell composition and clinicopathological factors in LUAD (left) and LUSQ (right). * p<0.05.

Fig. S7 Number and percentage of immune cell types, CD4+ T cell subsets, CD8 T+ cell subsets, and myeloid cell type in respective immune subtypes. (a–d) Cell density and %CD45 of immune cell type (a), %CD4 of CD4+ T cell subset (b), %CD8 of CD8+ T cell subset (c), and %myeloid of myeloid cell type (d) are presented in respective immune subtypes of LUAD and LUSQ. ns; not significant. * p<0.05. **P<0.01. ***P<0.001. ****P<0.0001.

Fig. S8 IHC of LUAD and LUSQ tissues of a representative case from immune subtypes. The antibodies against CD20 and FOXP3 are used in LUAD (a) and LUSQ tissues (b).

Fig. S9 CD33 IHC of LUAD and LUSQ tissues of a representative case from immune subtypes. (a) LUAD. (b)LUSQ. The number is the ratio of CD33 positive staining area per tissue area (percentage).

Fig. S10 Characterization of histopathological factors in respective immune subtypes of LUAD, LUSQ, and background NAT tissues. Ly: lymphatic vessel invasion, v: vascular invasion, pl; pleural invasion, pm: pulmonary metastasis, pT: tumor size, N: lymph node metastasis, M: distant metastasis. In NATs, fibrosis and frequencies of lymphocytes, neutrophils, and macrophages were assessed. The percentage of each classification in immune subtype was plotted in LUAD (a) and LUSQ (b) with NAT. The number of patients is specified in each column.

Fig. S11 Percentage of PD-1+ CD8 T+ and PD-L1+ CD8 T+ cells in respective immune subtypes. (a, b) Data were presented as a percentage of PD-1+ cells (a) and PD-L1+ cells (b) per indicated CD8+ T cell subset in LUAD and LUSQ. % naïve CD8; percentage of PD-1+ or PD-L1+ naïve CD8+ T cell subset per total of naïve CD8+ T cells. % CM CD8; percentage of PD-1+ or PD-L1+ CM CD8+ T cell subset per total of CM CD8+ T cells. % EM CD8; percentage of PD-1+ or PD-L1+ EM CD8+ T cell subset per total of EM CD8+ T cells. % EMRA CD8; percentage of PD-1+ or PD-L1+ EMRA CD8+ T cell subset per total of EMRA CD8+ T cells. ns; not significant. * p<0.05. **P<0.01.

Fig. S12 Metabolomic profiling in respective immune subtypes. Box plots showing abundances of immune-related amino acids and metabolites in LUAD (n=29) (Cold; n=3, Myeloid; n=17, CD8; n=9) and LUSQ (n=24) (Cold; n=8, Myeloid; n=13, CD8; n=3). ns; not significant. * p<0.05. **P<0.01.

Fig. S13 Relationship between immune subtypes and molecular subtypes. The molecular subtypes were analyzed by unsupervised consensus clustering of RNA-seq. The percentage of each molecular subtype in the immune subtype is plotted. The number of patients is specified in each column.

Fig. S14 Activated and suppressed pathways identified by GSEA with GO gene set in respective immune subtypes. (a, b) Heatmap representing top scored pathways enriched in genes showing commonly increased and decreased expression in respective immune subtypes in LUAD (a) and LUSQ (b). Top signaling pathways for genes showing increased expression are presented in red and those for genes showing decreased expression are presented in blue. (c, d) Running enrichment score with GO gene set of blood vessel morphogenesis, epidermis development, keratinocyte differentiation, and T cell activation signatures as the activated pathways in respective immune subtypes of LUAD (c) and LUSQ (d).

Fig. S15 Hazard ratios with deviation for specific signatures detected in the myeloid subtype of LUAD. The relationship between increased and decreased expression of signatures and event-free survival using transcriptome data from TCGA NSCLC was plotted. (a) hallmark gene set. (b) GO set.

Fig. S16 Boxplot showing the total number of non-synonymous mutations in respective immune subtypes. Non-synonymous mutations were calculated from WES data. ns; not significant.

Fig. S17 The relationship between TERT amplification and immune cell types in LUSQ. The correlations of the degree of TERT amplification with %CD45 of T, CD8+ T, mMDSC, and macrophage cells are plotted.

Table S1 Patient characteristics

Table S2 Antibodies for FACS

Table S3 Antibodies for IHC
