# Supplementary material for: Tumor-infiltrating Leukocyte Profiling Defines Three Immune Subtypes of NSCLC with Distinct Signaling Pathways and Genetic Alterations
Source: Cancer Res Commun. 2023 Jun 13;3(6):1026–40. doi: 10.1158/2767-9764.CRC-22-0415 (PMC10263066; doi:10.1158/2767-9764.CRC-22-0415)
Supplement: Fig. S1 — The number of patients and analyses in LUAD and LUSQ. The immunohistochemistry of tissue microarray, whole exome sequencing, TCR repertoire analysis, and metabolome analysis were performed in the cases FCM was conducted. [file crc-22-0415-s01.pdf]

Fig. S1

Adenocarcinoma; Tumor (n=155), non-tumor lung (n=117)

Tumor

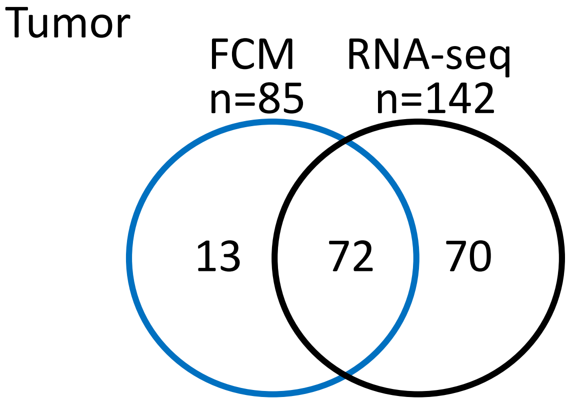

Cases in which FCM was performed (n=85)

	Done	Not-done
IHC	65	20
WES	69	16
TCR repertoire	55	30
Metabolome	29	56

FCM; flow cytometry
IHC; immunohistochemistry
WES; whole exome seq
TCR; T cell receptor

Squamous cell carcinoma; Tumor (n=80), non-tumor lung (n=65)

Tumor

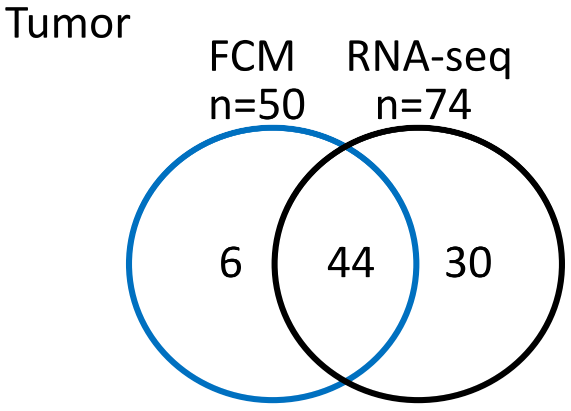

Cases in which FCM was performed (n=50)

	Done	Not-done
IHC	44	6
WES	45	5
TCR repertoire	36	14
Metabolome	24	26

Other lung cancer ; Tumor (n=46), non-tumor lung (n=37)

Tumor

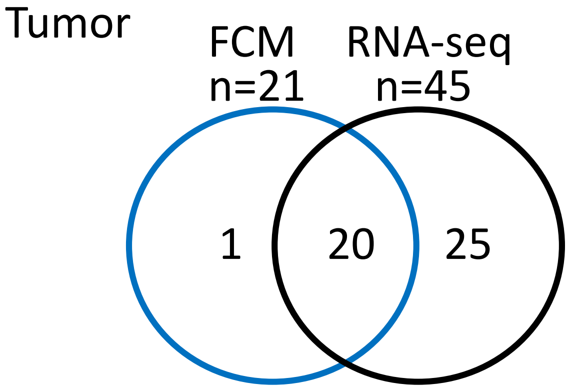

Figure S1. The number of patients and analyses in LUAD and LUSQ. The immunohistochemistry of tissue microarray, whole exome sequencing, TCR repertoire analysis, and metabolome analysis were performed in the cases FCM was conducted.
