# Supplementary material for: Tumor-infiltrating Leukocyte Profiling Defines Three Immune Subtypes of NSCLC with Distinct Signaling Pathways and Genetic Alterations
Source: Cancer Res Commun. 2023 Jun 13;3(6):1026–40. doi: 10.1158/2767-9764.CRC-22-0415 (PMC10263066; doi:10.1158/2767-9764.CRC-22-0415)
Supplement: Fig. S2 — Representative polychromatic dot plots demonstrating the gating strategy employed to identify the immune cell profile of TILs in NSCLC. Starting at the top left, the initial gate was to obtain live cells; next, leukocytes (CD45+) and epithelial cells (CD326+) were gated; finally, CD3+ cells and CD3- cells were separated. For CD3+ cells, a size gate was applied to identify NKT cells (CD3+ CD56+), CD3+CD4+ and CD3+CD8+ T cells. T cell subsets are displayed, including naïve (CD45RA+CD197+), central memory (CM: CD45RA-CD197+), effector memory (EM: CD45RA-CD197-), and effector memory cells re-expressing CD45RA (EMRA: CD45RA+CD197-). FOXP3-expressing CD4+ T cells were gated on CD4+ T cells: naïve Treg (Fr. I: CD45RA+FOXP3lo), effector Treg (Fr. II: CD45RA-FOXP3hi), and non-Treg (Fr. III: CD45RA-FOXP3lo). For CD3- cells, a size gate was applied to identify B (CD3-CD19+) and NK cells (CD3-CD56+). The myeloid cell lineage was also gated on CD3- cells: conventional DC (HLA-DR+CD11c+), plasmacytoid DC (HLA-DR+CD11c-CD123+), macrophage (CD68+SSChi) and monocytic MDSC (HLA-DRlo CD14+CD11b+CD33+). Ki-67-expressing cells were gated in CD8+NKT, CD4+NKT, CD4+T (NT, CM, EM, EMRA), CD8+T (NT, CM, EM, EMRA), Treg, B, NK, and mMDSC. PD-1-expressing cells were gated in CD4+T (naive, CM, EM, EMRA), CD8+T (naive, CM, EM, EMRA) and Treg cells. PD-L1-expressing cells were gated in CD8+T (NT, CM, EM, EMRA), and mMDSC. CTLA-4-expressing cells were gated in Treg cells. [file crc-22-0415-s02.pdf]

Fig. S2

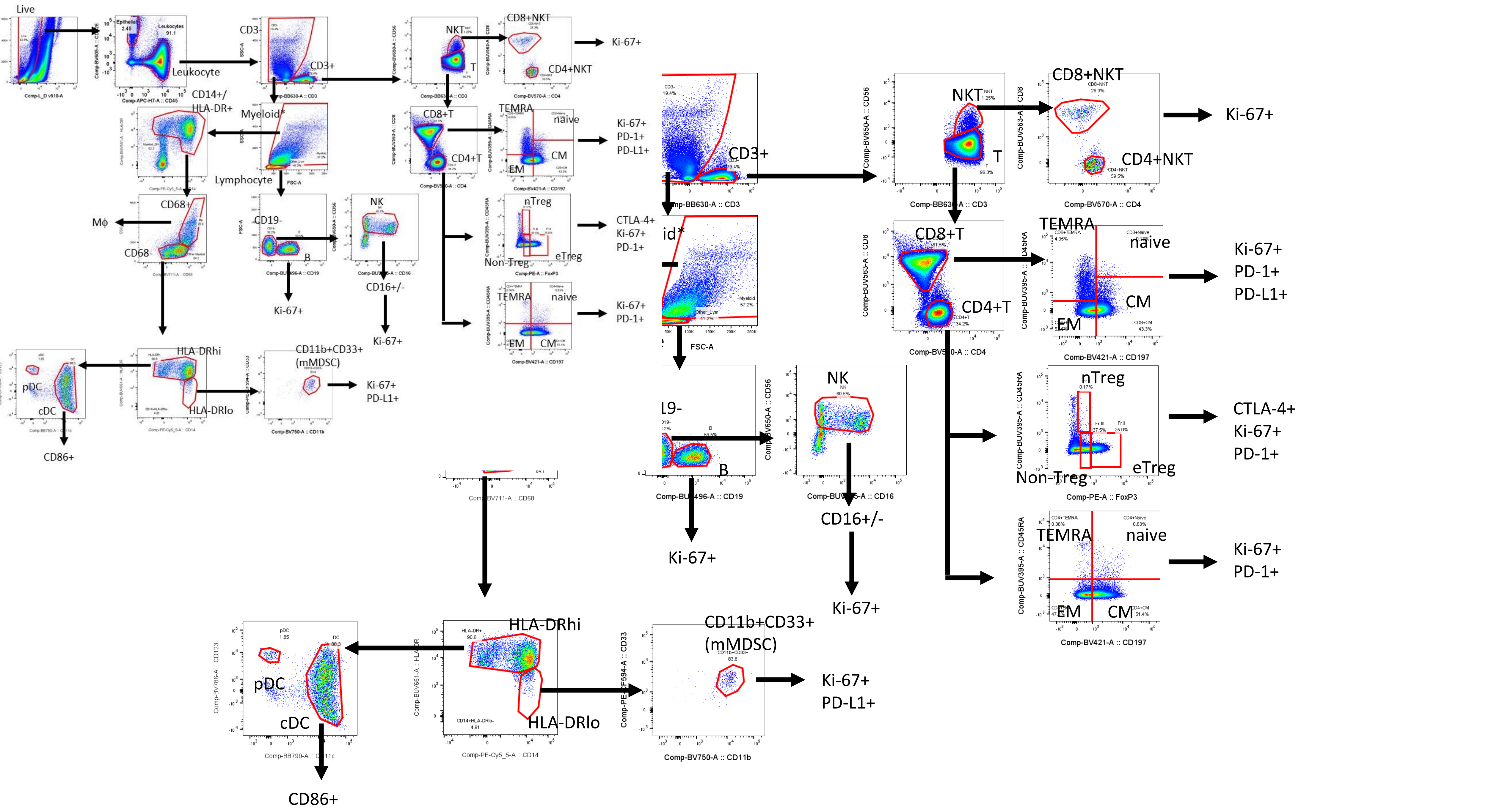

Figure S2. Representative polychromatic dot plots demonstrating the gating strategy employed to identify the immune cell profile of TILs in NSCLC. Starting at the top left, the initial gate was to obtain live cells; next, leukocytes ($CD45^{+}$) and epithelial cells ($CD326^{+}$) were gated; finally, $CD3^{+}$ cells and $CD3^{-}$ cells were separated. For $CD3^{+}$ cells, a size gate was applied to identify NKT cells ($CD3^{+}$ $CD56^{+}$), $CD3^{+}CD4^{+}$ and $CD3^{+}CD8^{+}$ T cells. T cell subsets are displayed, including naïve ($CD45RA^{+}CD197^{+}$), central memory (CM: $CD45RA^{-}CD197^{+}$), effector memory (EM: $CD45RA^{-}CD197^{-}$), and effector memory cells re-expressing CD45RA (EMRA: $CD45RA^{+}CD197^{-}$). FOXP3-expressing $CD4^{+}$ T cells were gated on $CD4^{+}$ T cells: naïve Treg (Fr. I: $CD45RA^{+}FOXP3^{lo}$), effector Treg (Fr. II: $CD45RA^{-}FOXP3^{hi}$), and non-Treg (Fr. III: $CD45RA^{-}FOXP3^{lo}$). For $CD3^{-}$ cells, a size gate was applied to identify B ($CD3^{-}CD19^{+}$) and NK cells ($CD3^{-}CD56^{+}$). The myeloid cell lineage was also gated on $CD3^{-}$ cells: conventional DC ($HLA\text{-}DR^{+}CD11c^{+}$), plasmacytoid DC ($HLA\text{-}DR^{+}CD11c^{-}$ $CD123^{+}$), macrophage ($CD68^{+}SSC^{hi}$) and monocytic MDSC ($HLA\text{-}DR^{lo}$ $CD14^{+}CD11b^{+}CD33^{+}$). Ki-67-expressing cells were gated in $CD8^{+}$NKT, $CD4^{+}$NKT, $CD4^{+}$T (NT, CM, EM, EMRA), $CD8^{+}$T (NT, CM, EM, EMRA), Treg, B, NK, and mMDSC. PD-1-expressing cells were gated in $CD4^{+}$T (naive, CM, EM, EMRA), $CD8^{+}$T (naive, CM, EM, EMRA) and Treg cells. PD-L1-expressing cells were gated in $CD8^{+}$T (NT, CM, EM, EMRA), and mMDSC. CTLA-4-expressing cells were gated in Treg cells.
