# Supplementary material for: Tumor-infiltrating Leukocyte Profiling Defines Three Immune Subtypes of NSCLC with Distinct Signaling Pathways and Genetic Alterations
Source: Cancer Res Commun. 2023 Jun 13;3(6):1026–40. doi: 10.1158/2767-9764.CRC-22-0415 (PMC10263066; doi:10.1158/2767-9764.CRC-22-0415)
Supplement: Fig. S3 — Number of each immune cell type, CD4+ T cell subset, and CD8 T+ cell subset by FCM from lung cancer and NATs. (a) Cell density of each immune cell type. Data are presented as the number of indicated immune cells per gram of NAT, LUAD, LUSQ, and other types of lung cancer tissues. (b, c) Density of CD4+ T cell subset (b) and CD8+ T cell subset (c). ns; not significant. * p<0.05. **P<0.01. ***P<0.001. ****P<0.0001. [file crc-22-0415-s03.pdf]

Fig. S3

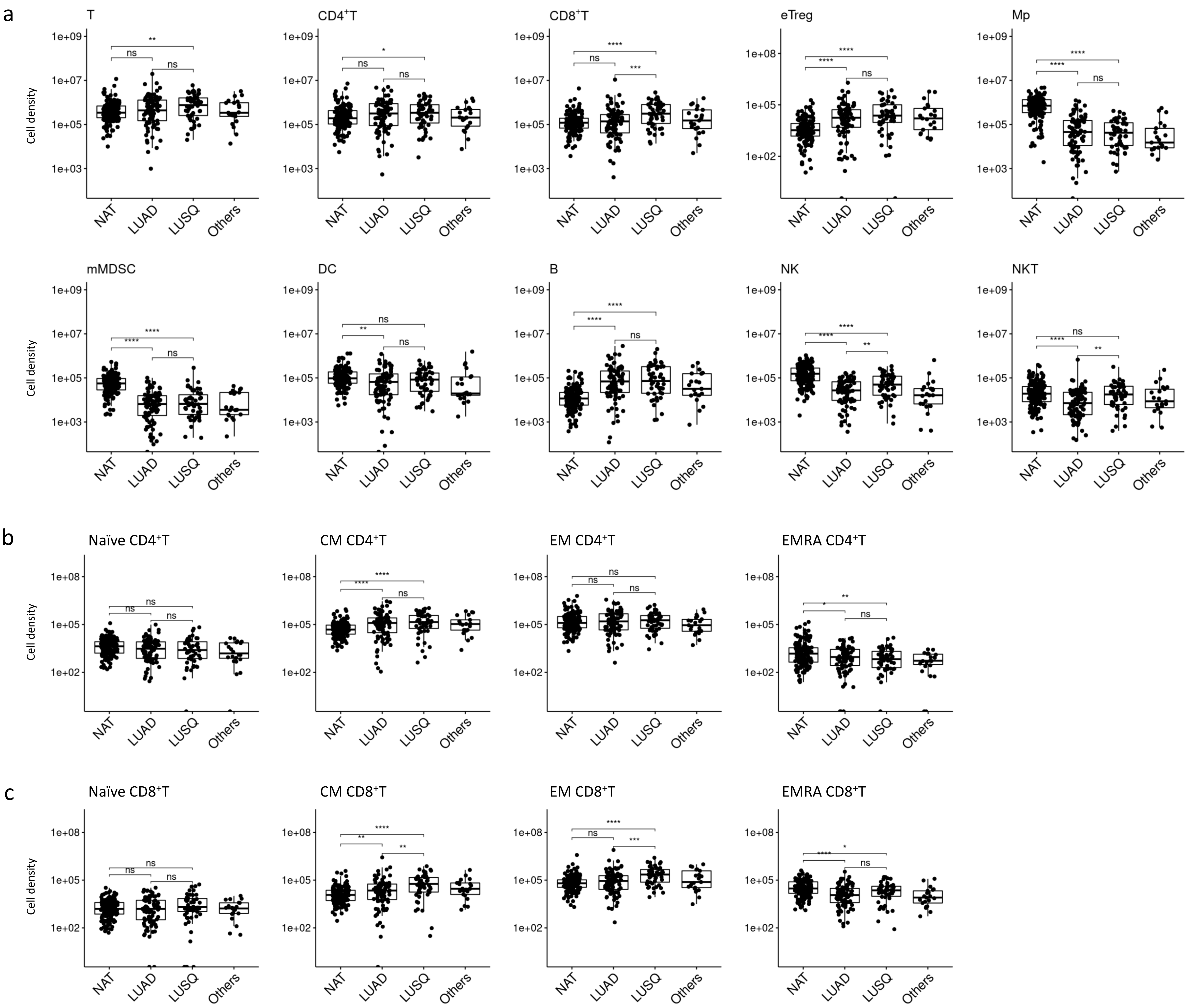

Figure S3. Number of each immune cell type, $CD4^+$ T cell subset, and CD8 T^+ cell subset by FCM from lung cancer and NATs. (a) Cell density of each immune cell type. Data are presented as the number of indicated immune cells per gram of NAT, LUAD, LUSQ, and other types of lung cancer tissues. (b, c) Density of $CD4^+$ T cell subset (b) and $CD8^+$ T cell subset (c). ns; not significant. * p<0.05. **P<0.01. ***P<0.001. ****P<0.0001.
