# Supplementary material for: Tumor-infiltrating Leukocyte Profiling Defines Three Immune Subtypes of NSCLC with Distinct Signaling Pathways and Genetic Alterations
Source: Cancer Res Commun. 2023 Jun 13;3(6):1026–40. doi: 10.1158/2767-9764.CRC-22-0415 (PMC10263066; doi:10.1158/2767-9764.CRC-22-0415)
Supplement: Fig. S4 — Relation of immune cell types with histological grade. LUAD (n=77) (well differentiated; n=2, moderately differentiated; n=41, poorly differentiated; n=34) and LUSQ (n=50) (well differentiated; n=8, moderately differentiated; n=32, poorly differentiated; n=10). (a–d) %CD45 of immune cell type (a), %CD4 of CD4+ T cell subset (b), %CD8 of CD8+ T cell subset (c), and %myeloid of myeloid cell type (d) are presented in well, moderately, and poorly differentiated LUAD and LUSQ. (e) %CD4 of CTLA-4+ eTregs in LUAD and LUSQ. (f) %CD8 of PD-1+ and PD-L1+ CM CD8+ T cells in LUAD and LUSQ. ns; not significant. * p<0.05. **P<0.01. [file crc-22-0415-s04.pdf]

Fig. S4

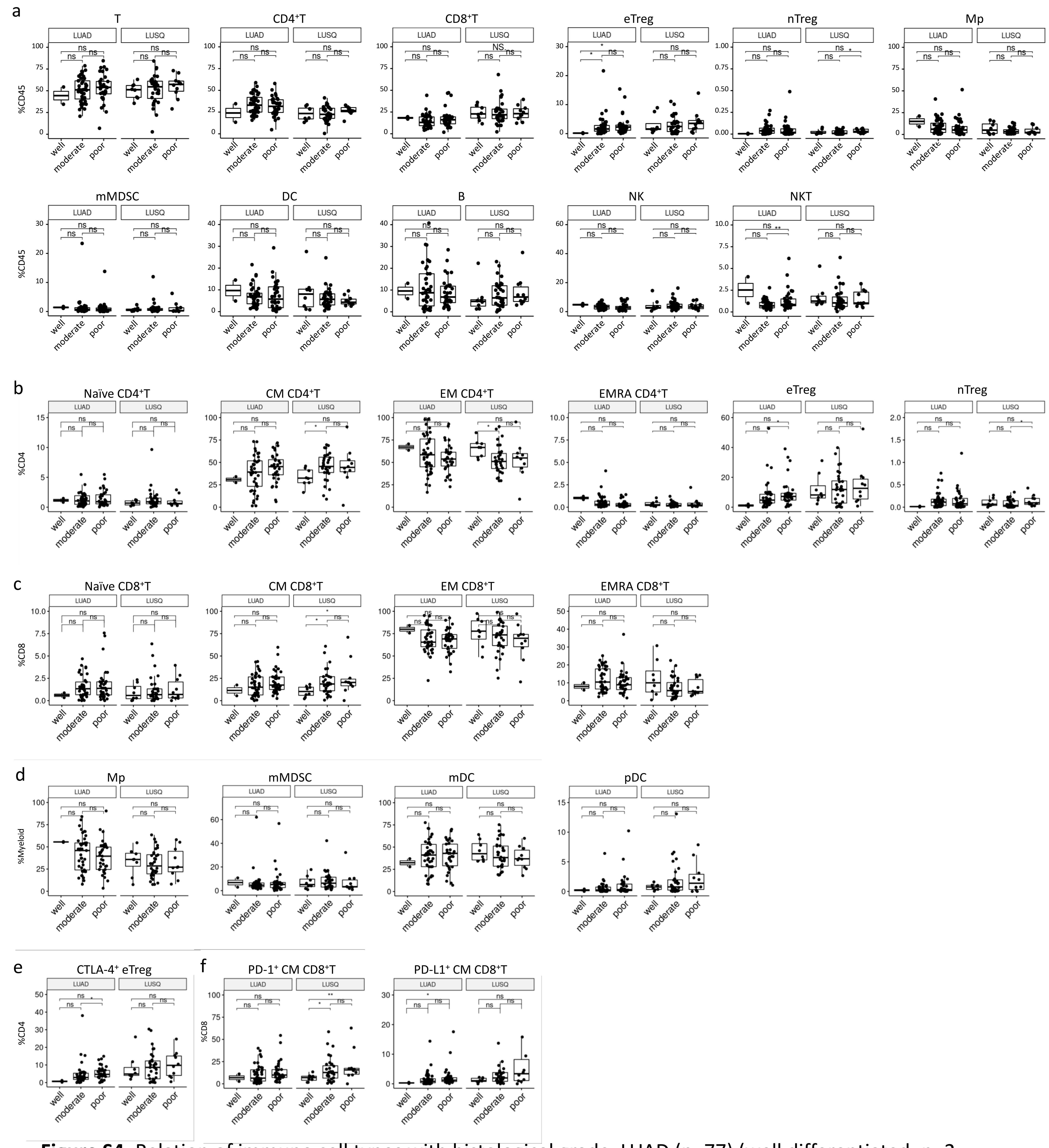

Figure S4. Relation of immune cell types with histological grade. LUAD (n=77) (well differentiated; n=2, moderately differentiated; n=41, poorly differentiated; n=34) and LUSQ (n=50) (well differentiated; n=8, moderately differentiated; n=32, poorly differentiated; n=10). (a–d) %CD45 of immune cell type (a), %CD4 of $CD4^+$ T cell subset (b), %CD8 of $CD8^+$ T cell subset (c), and %myeloid of myeloid cell type (d) are presented in well, moderately, and poorly differentiated LUAD and LUSQ. (e) %CD4 of CTLA-4^+ eTregs in LUAD and LUSQ. (f) %CD8 of PD-1^+ and PD-$L1^+$ CM $CD8^+$ T cells in LUAD and LUSQ. ns; not significant. * $p<0.05$. ** $P<0.01$.
