# Supplementary material for: Tumor-infiltrating Leukocyte Profiling Defines Three Immune Subtypes of NSCLC with Distinct Signaling Pathways and Genetic Alterations
Source: Cancer Res Commun. 2023 Jun 13;3(6):1026–40. doi: 10.1158/2767-9764.CRC-22-0415 (PMC10263066; doi:10.1158/2767-9764.CRC-22-0415)
Supplement: Fig. S5 — Relation of immune cell types with WHO classification. LUAD (n=82) (lepidic; n=2, acinar; n=7, papillary; n=39, micropapillary; n=11, solid; n=15, IMA; n=8) and LUSQ (n=50) (non-keratinizing; n=6, keratinizing; n=42, basaloid; n=2). (a–d) %CD45 of immune cell type (a), %CD4 of CD4+ T cell subset (b), %CD8 of CD8+ T cell subset (c), and %myeloid of myeloid cell type (d) are presented following the WHO classification of LUAD and LUSQ. ns; not significant. * p<0.05. **P<0.01. ***P<0.001. [file crc-22-0415-s05.pdf]

Fig. S5

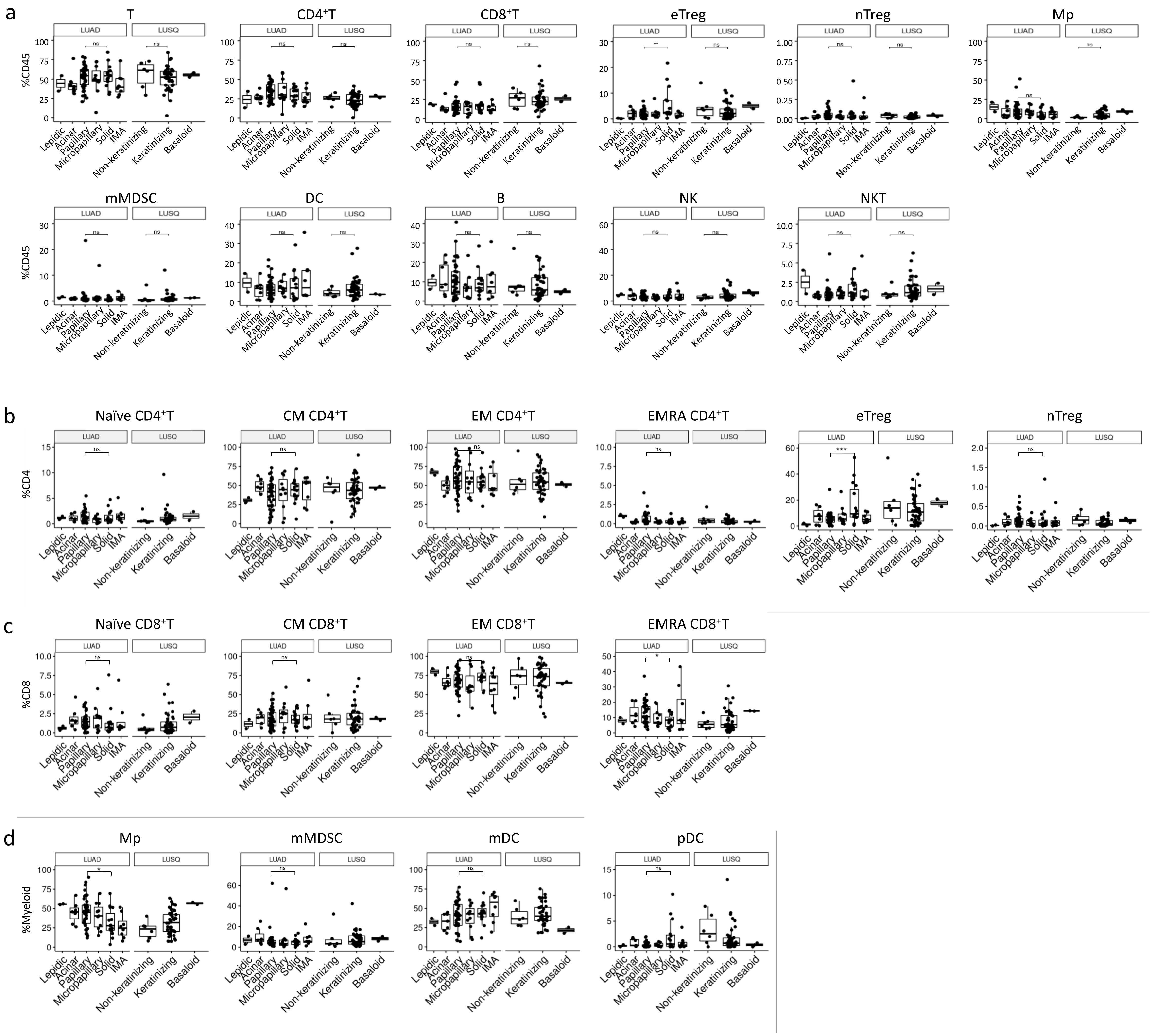

Figure S5. Relation of immune cell types with WHO classification. LUAD (n=82) (lepidic; n=2, acinar; n=7, papillary; n=39, micropapillary; n=11, solid; n=15, IMA; n=8) and LUSQ (n=50) (non-keratinizing; n=6, keratinizing; n=42, basaloid; n=2). (a–d) %CD45 of immune cell type (a), %CD4 of $CD4^+$ T cell subset (b), %CD8 of $CD8^+$ T cell subset (c), and %myeloid of myeloid cell type (d) are presented following the WHO classification of LUAD and LUSQ. ns; not significant. * p<0.05. **P<0.01. ***P<0.001.
