# Supplementary material for: Tumor-infiltrating Leukocyte Profiling Defines Three Immune Subtypes of NSCLC with Distinct Signaling Pathways and Genetic Alterations
Source: Cancer Res Commun. 2023 Jun 13;3(6):1026–40. doi: 10.1158/2767-9764.CRC-22-0415 (PMC10263066; doi:10.1158/2767-9764.CRC-22-0415)
Supplement: Fig. S6 — Relationship between the number of immune cell types and clinicopathological factors. Cells in the matrix represent the 1-Pearson correlation coefficient between cell density of the indicated immune cell composition and clinicopathological factors in LUAD (left) and LUSQ (right). * p<0.05. [file crc-22-0415-s06.pdf]

Fig. S6

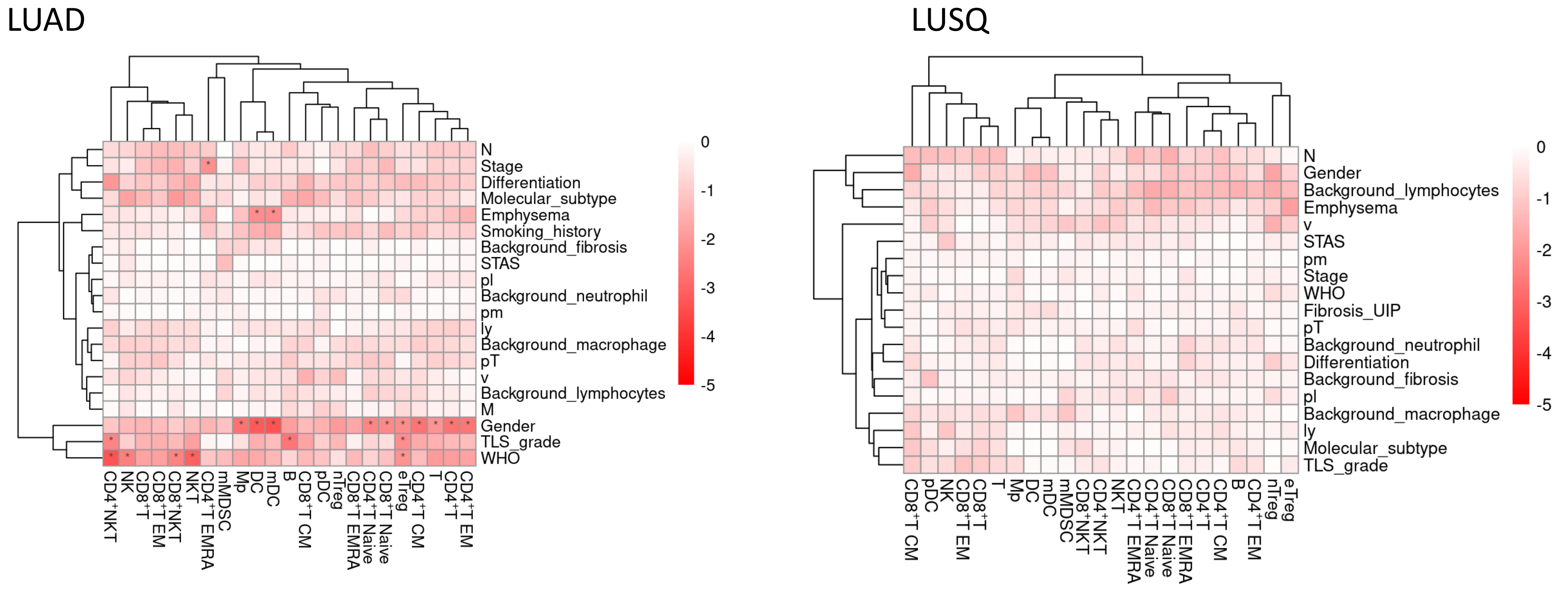

Figure S6. Relationship between the number of immune cell types and clinicopathological factors. Cells in the matrix represent the 1-Pearson correlation coefficient between cell density of the indicated immune cell composition and clinicopathological factors in LUAD (left) and LUSQ (right). * $p<0.05$.
