# Supplementary material for: Tumor-infiltrating Leukocyte Profiling Defines Three Immune Subtypes of NSCLC with Distinct Signaling Pathways and Genetic Alterations
Source: Cancer Res Commun. 2023 Jun 13;3(6):1026–40. doi: 10.1158/2767-9764.CRC-22-0415 (PMC10263066; doi:10.1158/2767-9764.CRC-22-0415)
Supplement: Fig. S7 — Number and percentage of immune cell types, CD4+ T cell subsets, CD8 T+ cell subsets, and myeloid cell type in respective immune subtypes. (a–d) Cell density and %CD45 of immune cell type (a), %CD4 of CD4+ T cell subset (b), %CD8 of CD8+ T cell subset (c), and %myeloid of myeloid cell type (d) are presented in respective immune subtypes of LUAD and LUSQ. ns; not significant. * p<0.05. **P<0.01. ***P<0.001. ****P<0.0001. [file crc-22-0415-s07.pdf]

Fig. S7

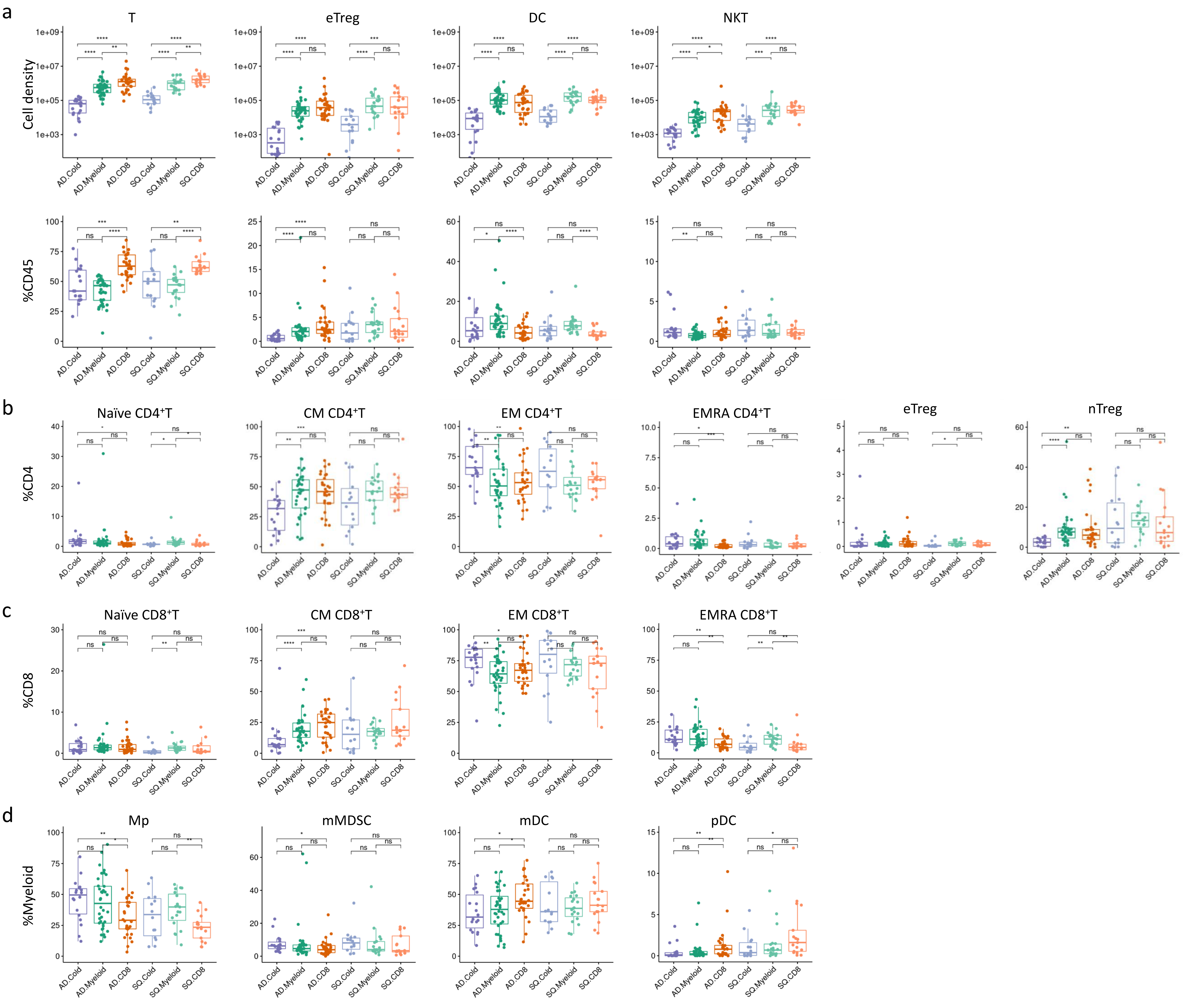

Figure S7. Number and percentage of immune cell types, CD4^{+} T cell subsets, CD8 T^{+} cell subsets, and myeloid cell type in respective immune subtypes. (a–d) Cell density and %CD45 of immune cell type (a), %CD4 of CD4^{+} T cell subset (b), %CD8 of CD8^{+} T cell subset (c), and %myeloid of myeloid cell type (d) are presented in respective immune subtypes of LUAD and LUSQ. ns; not significant. * $p<0.05$. ** $P<0.01$. *** $P<0.001$. **** $P<0.0001$.
