# Supplementary material for: Tumor-infiltrating Leukocyte Profiling Defines Three Immune Subtypes of NSCLC with Distinct Signaling Pathways and Genetic Alterations
Source: Cancer Res Commun. 2023 Jun 13;3(6):1026–40. doi: 10.1158/2767-9764.CRC-22-0415 (PMC10263066; doi:10.1158/2767-9764.CRC-22-0415)
Supplement: Fig. S8 — IHC of LUAD and LUSQ tissues of a representative case from immune subtypes. The antibodies against CD20 and FOXP3 are used in LUAD (a) and LUSQ tissues (b). [file crc-22-0415-s08.pdf]

Fig. S8

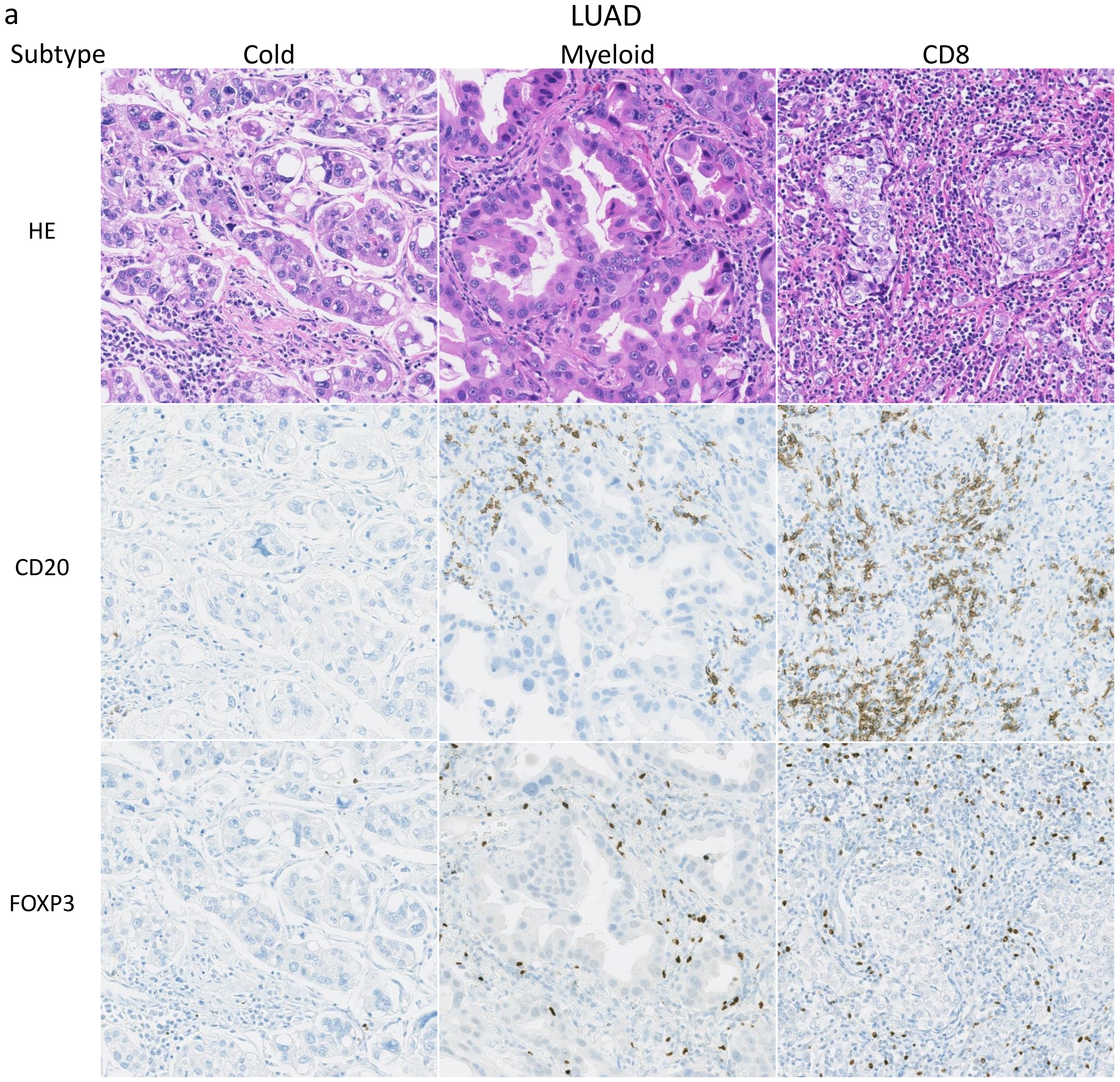
a
LUAD
Subtype
Cold
Myeloid
CD8
HE
CD20
FOXP3

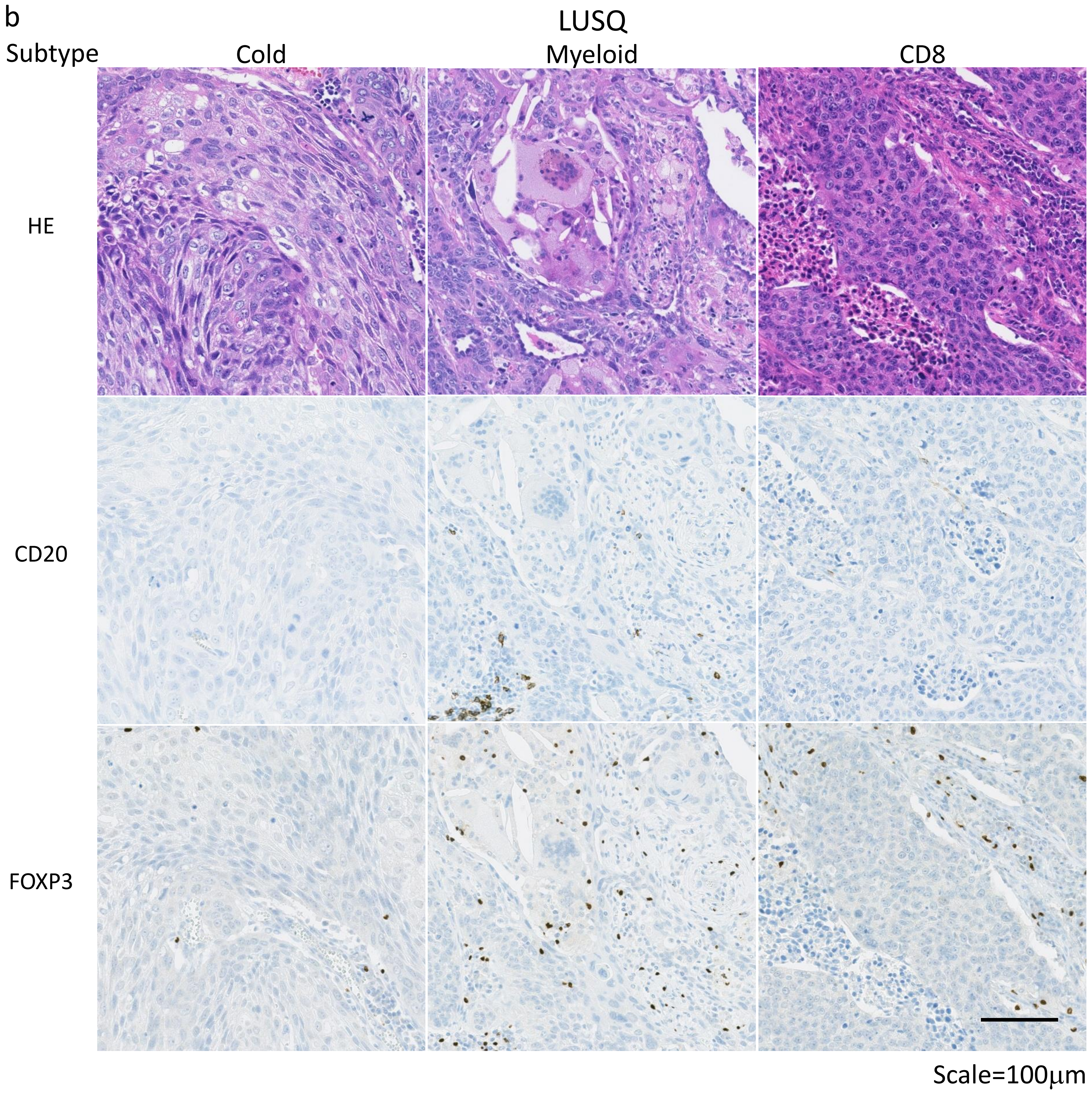
b
LUSQ
Subtype
Cold
Myeloid
CD8
HE
CD20
FOXP3
Scale=100μm

Figure S8. IHC of LUAD and LUSQ tissues of a representative case from immune subtypes. The antibodies against CD20 and FOXP3 are used in LUAD (a) and LUSQ tissues (b).
