# Supplementary material for: Tumor-infiltrating Leukocyte Profiling Defines Three Immune Subtypes of NSCLC with Distinct Signaling Pathways and Genetic Alterations
Source: Cancer Res Commun. 2023 Jun 13;3(6):1026–40. doi: 10.1158/2767-9764.CRC-22-0415 (PMC10263066; doi:10.1158/2767-9764.CRC-22-0415)
Supplement: Fig. S9 — CD33 IHC of LUAD and LUSQ tissues of a representative case from immune subtypes. (a) LUAD. (b)LUSQ. The number is the ratio of CD33 positive staining area per tissue area (percentage). [file crc-22-0415-s09.pdf]

Fig. S9

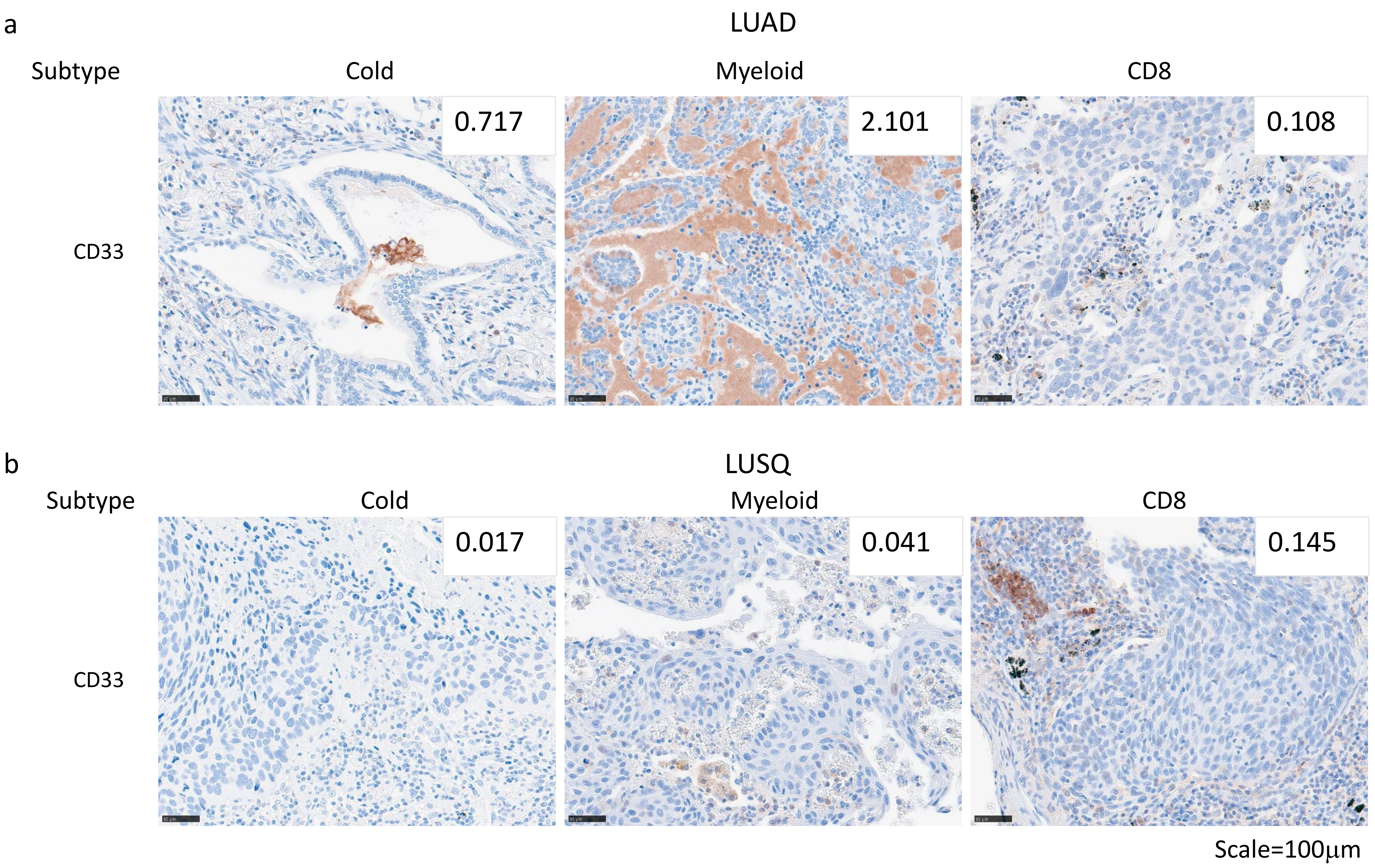

Figure S9. CD33 IHC of LUAD and LUSQ tissues of a representative case from immune subtypes. (a) LUAD. (b)LUSQ. The number is the ratio of CD33 positive staining area per tissue area (percentage).
