# Supplementary material for: Tumor-infiltrating Leukocyte Profiling Defines Three Immune Subtypes of NSCLC with Distinct Signaling Pathways and Genetic Alterations
Source: Cancer Res Commun. 2023 Jun 13;3(6):1026–40. doi: 10.1158/2767-9764.CRC-22-0415 (PMC10263066; doi:10.1158/2767-9764.CRC-22-0415)
Supplement: Fig. S10 — Characterization of histopathological factors in respective immune subtypes of LUAD, LUSQ, and background NAT tissues. Ly: lymphatic vessel invasion, v: vascular invasion, pl; pleural invasion, pm: pulmonary metastasis, pT: tumor size, N: lymph node metastasis, M: distant metastasis. In NATs, fibrosis and frequencies of lymphocytes, neutrophils, and macrophages were assessed. The percentage of each classification in immune subtype was plotted in LUAD (a) and LUSQ (b) with NAT. The number of patients is specified in each column. [file crc-22-0415-s10.pdf]

Fig. S10

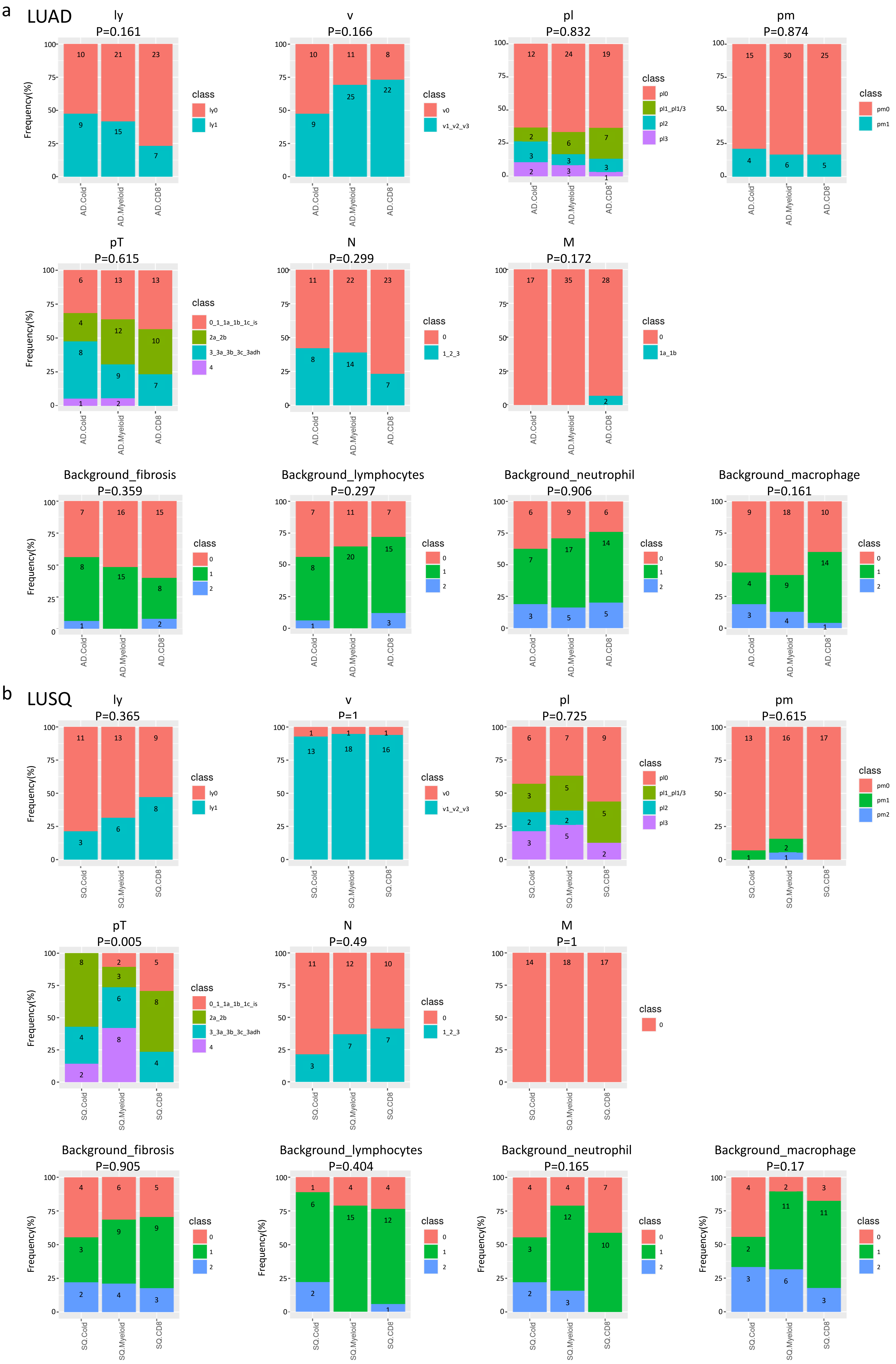
a
LUAD
ly
P=0.161
v
P=0.166
pl
P=0.832
pm
P=0.874
pT
P=0.615
N
P=0.299
M
P=0.172
Background_fibrosis
P=0.359
Background_lymphocytes
P=0.297
Background_neutrophil
P=0.906
Background_macrophage
P=0.161
Frequency(%)
class
ly0
ly1
v0
v1_v2_v3
pl0
pl1_pl1/3
pl2
pl3
pm0
pm1
0_1_1a_1b_1c_is
2a_2b
3_3a_3b_3c_3adh
4
0
1_2_3
1a_1b
0
1
2
AD.Cold
AD.Myeloid
AD.CD8
b
LUSQ
ly
P=0.365
v
P=1
pl
P=0.725
pm
P=0.615
pT
P=0.005
N
P=0.49
M
P=1
Background_fibrosis
P=0.905
Background_lymphocytes
P=0.404
Background_neutrophil
P=0.165
Background_macrophage
P=0.17
pm2
SQ.Cold
SQ.Myeloid
SQ.CD8

Figure S10. Characterization of histopathological factors in respective immune subtypes of LUAD, LUSQ, and background NAT tissues. Ly: lymphatic vessel invasion, v: vascular invasion, pl; pleural invasion, pm: pulmonary metastasis, pT: tumor size, N: lymph node metastasis, M: distant metastasis. In NATs, fibrosis and frequencies of lymphocytes, neutrophils, and macrophages were assessed. The percentage of each classification in immune subtype was plotted in LUAD (a) and LUSQ (b) with NAT. The number of patients is specified in each column.
