# Supplementary material for: Tumor-infiltrating Leukocyte Profiling Defines Three Immune Subtypes of NSCLC with Distinct Signaling Pathways and Genetic Alterations
Source: Cancer Res Commun. 2023 Jun 13;3(6):1026–40. doi: 10.1158/2767-9764.CRC-22-0415 (PMC10263066; doi:10.1158/2767-9764.CRC-22-0415)
Supplement: Fig. S11 — Percentage of PD-1+ CD8 T+ and PD-L1+ CD8 T+ cells in respective immune subtypes. (a, b) Data were presented as a percentage of PD-1+ cells (a) and PD-L1+ cells (b) per indicated CD8+ T cell subset in LUAD and LUSQ. % naïve CD8; percentage of PD-1+ or PD-L1+ naïve CD8+ T cell subset per total of naïve CD8+ T cells. % CM CD8; percentage of PD-1+ or PD-L1+ CM CD8+ T cell subset per total of CM CD8+ T cells. % EM CD8; percentage of PD-1+ or PD-L1+ EM CD8+ T cell subset per total of EM CD8+ T cells. % EMRA CD8; percentage of PD-1+ or PD-L1+ EMRA CD8+ T cell subset per total of EMRA CD8+ T cells. ns; not significant. * p<0.05. **P<0.01. [file crc-22-0415-s11.pdf]

Fig. S11

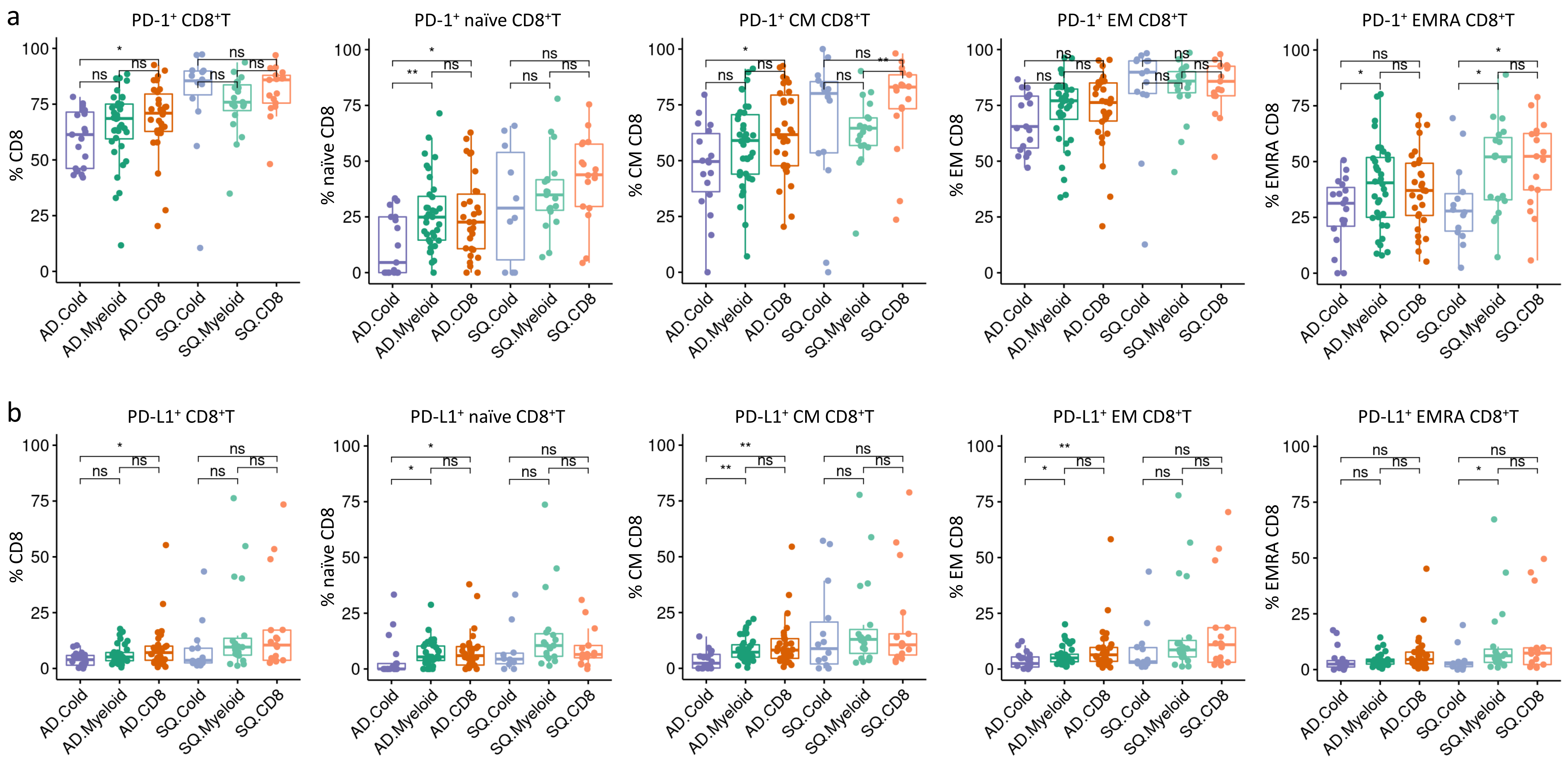

Figure S11. Percentage of PD-1⁺ CD8 T⁺ and PD-L1⁺ CD8 T⁺ cells in respective immune subtypes. (a, b) Data were presented as a percentage of PD-1⁺ cells (a) and PD-L1⁺ cells (b) per indicated CD8⁺ T cell subset in LUAD and LUSQ. % naïve CD8; percentage of PD-1⁺ or PD-L1⁺ naïve CD8⁺ T cell subset per total of naïve CD8⁺ T cells. % CM CD8; percentage of PD-1⁺ or PD-L1⁺ CM CD8⁺ T cell subset per total of CM CD8⁺ T cells. % EM CD8; percentage of PD-1⁺ or PD-L1⁺ EM CD8⁺ T cell subset per total of EM CD8⁺ T cells. % EMRA CD8; percentage of PD-1⁺ or PD-L1⁺ EMRA CD8⁺ T cell subset per total of EMRA CD8⁺ T cells. ns; not significant. * p<0.05. **P<0.01.
