# Supplementary material for: Tumor-infiltrating Leukocyte Profiling Defines Three Immune Subtypes of NSCLC with Distinct Signaling Pathways and Genetic Alterations
Source: Cancer Res Commun. 2023 Jun 13;3(6):1026–40. doi: 10.1158/2767-9764.CRC-22-0415 (PMC10263066; doi:10.1158/2767-9764.CRC-22-0415)
Supplement: Fig. S12 — Metabolomic profiling in respective immune subtypes. Box plots showing abundances of immune-related amino acids and metabolites in LUAD (n=29) (Cold; n=3, Myeloid; n=17, CD8; n=9) and LUSQ (n=24) (Cold; n=8, Myeloid; n=13, CD8; n=3). ns; not significant. * p<0.05. **P<0.01. [file crc-22-0415-s12.pdf]

Fig. S12

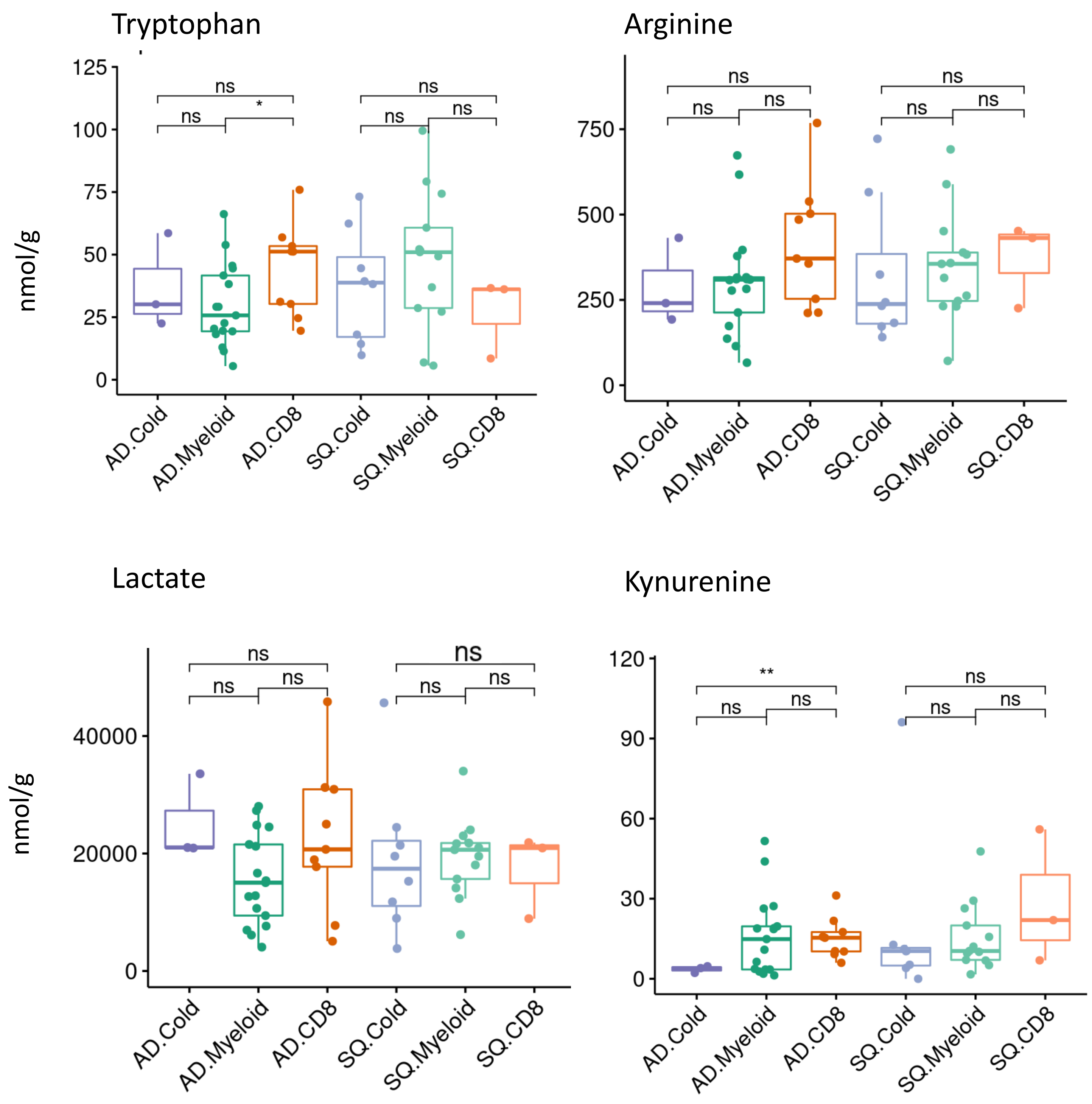

Figure S12. Metabolomic profiling in respective immune subtypes. Box plots showing abundances of immune-related amino acids and metabolites in LUAD (n=29) (Cold; n=3, Myeloid; n=17, CD8; n=9) and LUSQ (n=24) (Cold; n=8, Myeloid; n=13, CD8; n=3). ns; not significant. * p<0.05. **P<0.01.
