# Supplementary material for: Tumor-infiltrating Leukocyte Profiling Defines Three Immune Subtypes of NSCLC with Distinct Signaling Pathways and Genetic Alterations
Source: Cancer Res Commun. 2023 Jun 13;3(6):1026–40. doi: 10.1158/2767-9764.CRC-22-0415 (PMC10263066; doi:10.1158/2767-9764.CRC-22-0415)
Supplement: Fig. S13 — Relationship between immune subtypes and molecular subtypes. The molecular subtypes were analyzed by unsupervised consensus clustering of RNA-seq. The percentage of each molecular subtype in the immune subtype is plotted. The number of patients is specified in each column. [file crc-22-0415-s13.pdf]

Fig. S13

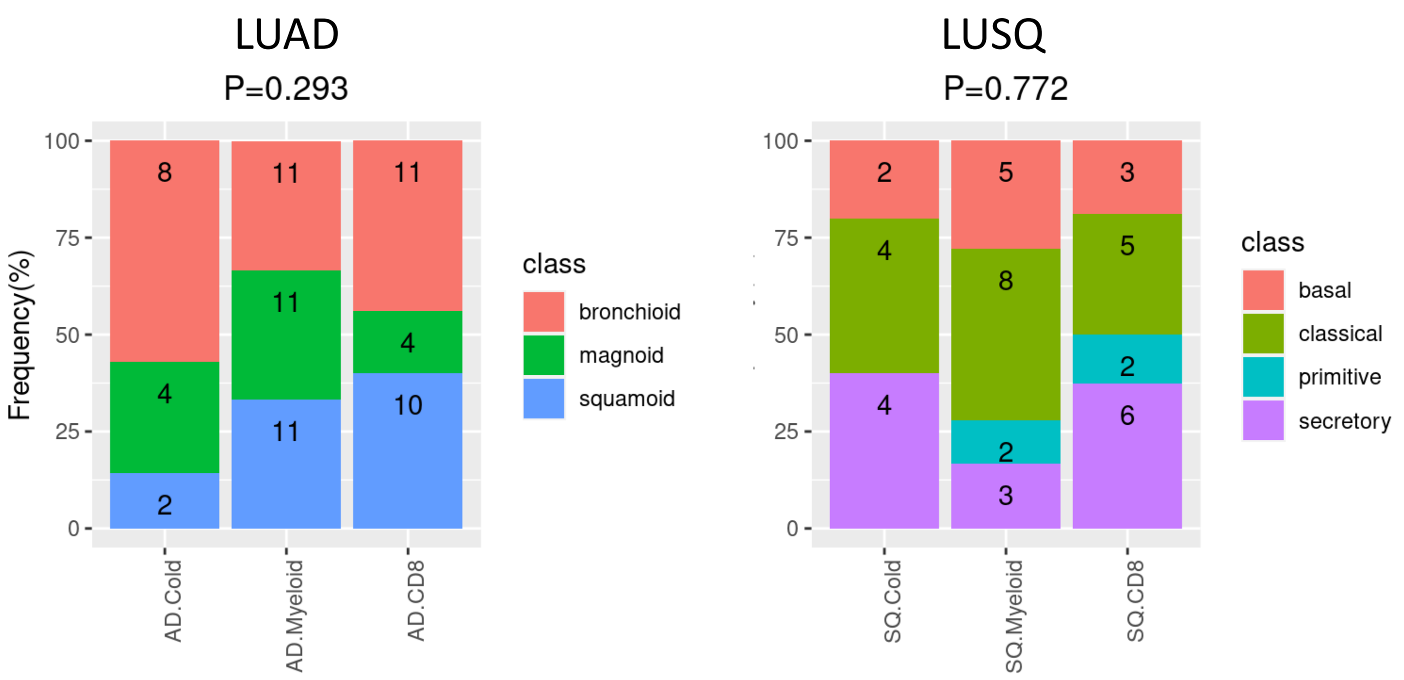

Figure S13. Relationship between immune subtypes and molecular subtypes. The molecular subtypes were analyzed by unsupervised consensus clustering of RNA-seq. The percentage of each molecular subtype in the immune subtype is plotted. The number of patients is specified in each column.
