# Supplementary material for: Tumor-infiltrating Leukocyte Profiling Defines Three Immune Subtypes of NSCLC with Distinct Signaling Pathways and Genetic Alterations
Source: Cancer Res Commun. 2023 Jun 13;3(6):1026–40. doi: 10.1158/2767-9764.CRC-22-0415 (PMC10263066; doi:10.1158/2767-9764.CRC-22-0415)
Supplement: Fig. S14 — Activated and suppressed pathways identified by GSEA with GO gene set in respective immune subtypes. (a, b) Heatmap representing top scored pathways enriched in genes showing commonly increased and decreased expression in respective immune subtypes in LUAD (a) and LUSQ (b). Top signaling pathways for genes showing increased expression are presented in red and those for genes showing decreased expression are presented in blue. (c, d) Running enrichment score with GO gene set of blood vessel morphogenesis, epidermis development, keratinocyte differentiation, and T cell activation signatures as the activated pathways in respective immune subtypes of LUAD (c) and LUSQ (d). [file crc-22-0415-s14.pdf]

Fig. S14

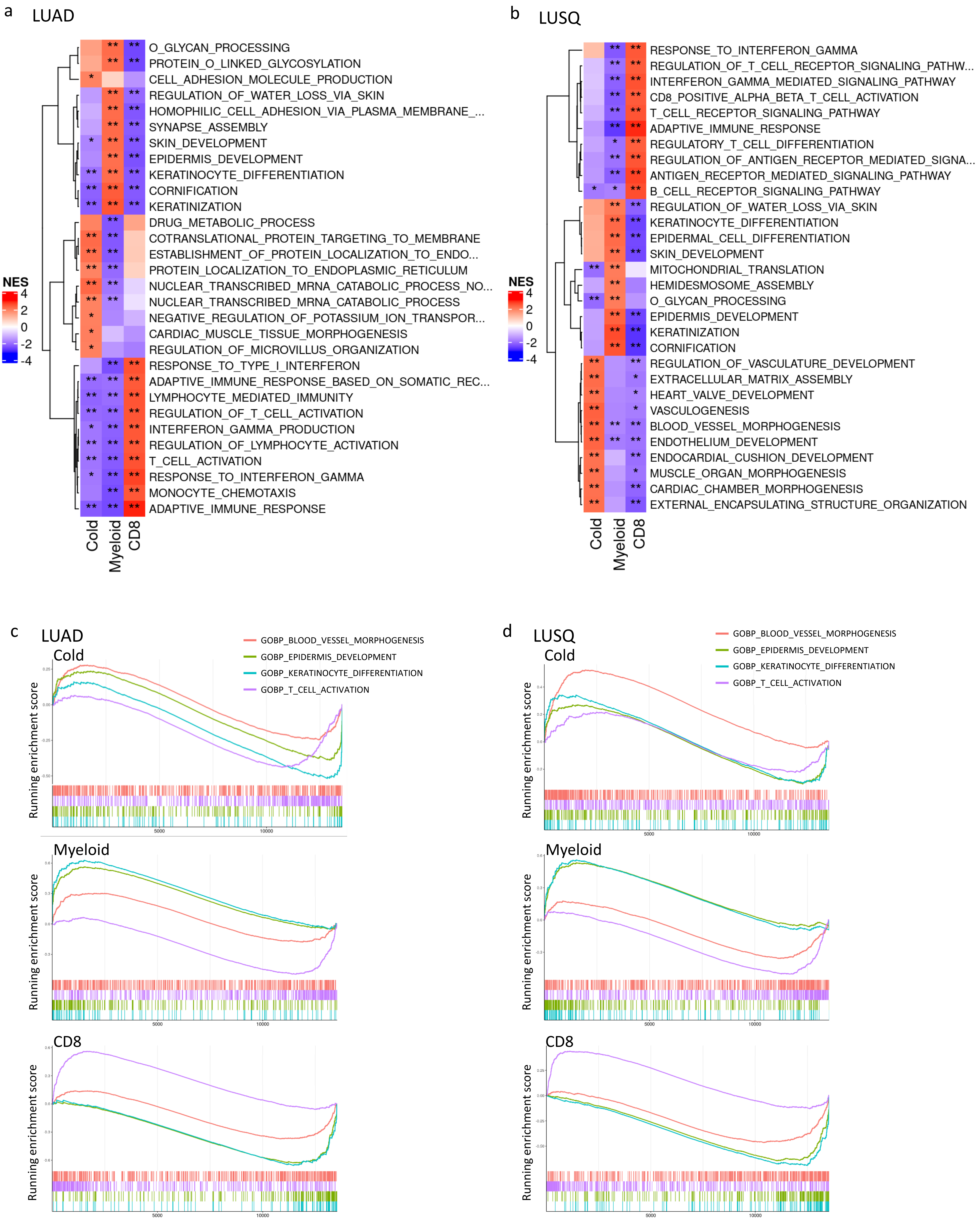
a
LUAD
O_GLYCAN_PROCESSING
PROTEIN_O_LINKED_GLYCOSYLATION
CELL_ADHESION_MOLECULE_PRODUCTION
REGULATION_OF_WATER_LOSS_VIA_SKIN
HOMOPHILIC_CELL_ADHESION_VIA_PLASMA_MEMBRANE_...
SYNAPSE_ASSEMBLY
SKIN_DEVELOPMENT
EPIDERMIS_DEVELOPMENT
KERATINOCYTE_DIFFERENTIATION
CORNIFICATION
KERATINIZATION
DRUG_METABOLIC_PROCESS
COTRANSLATIONAL_PROTEIN_TARGETING_TO_MEMBRANE
ESTABLISHMENT_OF_PROTEIN_LOCALIZATION_TO_ENDO...
PROTEIN_LOCALIZATION_TO_ENDOPLASMIC_RETICULUM
NUCLEAR_TRANSCRIBED_MRNA_CATABOLIC_PROCESS_NO...
NUCLEAR_TRANSCRIBED_MRNA_CATABOLIC_PROCESS
NEGATIVE_REGULATION_OF_POTASSIUM_ION_TRANSPOR...
CARDIAC_MUSCLE_TISSUE_MORPHOGENESIS
REGULATION_OF_MICROVILLUS_ORGANIZATION
RESPONSE_TO_TYPE_I_INTERFERON
ADAPTIVE_IMMUNE_RESPONSE_BASED_ON_SOMATIC_REC...
LYMPHOCYTE_MEDIATED_IMMUNITY
REGULATION_OF_T_CELL_ACTIVATION
INTERFERON_GAMMA_PRODUCTION
REGULATION_OF_LYMPHOCYTE_ACTIVATION
T_CELL_ACTIVATION
RESPONSE_TO_INTERFERON_GAMMA
MONOCYTE_CHEMOTAXIS
ADAPTIVE_IMMUNE_RESPONSE
NES
4
2
0
-2
-4
Cold
Myeloid
CD8
b
LUSQ
RESPONSE_TO_INTERFERON_GAMMA
REGULATION_OF_T_CELL_RECEPTOR_SIGNALING_PATHW...
INTERFERON_GAMMA_MEDIATED_SIGNALING_PATHWAY
CD8_POSITIVE_ALPHA_BETA_T_CELL_ACTIVATION
T_CELL_RECEPTOR_SIGNALING_PATHWAY
ADAPTIVE_IMMUNE_RESPONSE
REGULATORY_T_CELL_DIFFERENTIATION
REGULATION_OF_ANTIGEN_RECEPTOR_MEDIATED_SIGNA...
ANTIGEN_RECEPTOR_MEDIATED_SIGNALING_PATHWAY
B_CELL_RECEPTOR_SIGNALING_PATHWAY
REGULATION_OF_WATER_LOSS_VIA_SKIN
KERATINOCYTE_DIFFERENTIATION
EPIDERMAL_CELL_DIFFERENTIATION
SKIN_DEVELOPMENT
MITOCHONDRIAL_TRANSLATION
HEMIDESMOSOME_ASSEMBLY
O_GLYCAN_PROCESSING
EPIDERMIS_DEVELOPMENT
KERATINIZATION
CORNIFICATION
REGULATION_OF_VASCULATURE_DEVELOPMENT
EXTRACELLULAR_MATRIX_ASSEMBLY
HEART_VALVE_DEVELOPMENT
VASCULOGENESIS
BLOOD_VESSEL_MORPHOGENESIS
ENDOTHELIUM_DEVELOPMENT
ENDOCARDIAL_CUSHION_DEVELOPMENT
MUSCLE_ORGAN_MORPHOGENESIS
CARDIAC_CHAMBER_MORPHOGENESIS
EXTERNAL_ENCAPSULATING_STRUCTURE_ORGANIZATION
NES
4
2
0
-2
-4
Cold
Myeloid
CD8
c
LUAD
Cold
GOBP_BLOOD_VESSEL_MORPHOGENESIS
GOBP_EPIDERMIS_DEVELOPMENT
GOBP_KERATINOCYTE_DIFFERENTIATION
GOBP_T_CELL_ACTIVATION
Running enrichment score
Myeloid
CD8
5000
10000
d
LUSQ
Cold
GOBP_BLOOD_VESSEL_MORPHOGENESIS
GOBP_EPIDERMIS_DEVELOPMENT
GOBP_KERATINOCYTE_DIFFERENTIATION
GOBP_T_CELL_ACTIVATION
Running enrichment score
Myeloid
CD8
5000
10000

Figure S14. Activated and suppressed pathways identified by GSEA with GO gene set in respective immune subtypes. (a, b) Heatmap representing top scored pathways enriched in genes showing commonly increased and decreased expression in respective immune subtypes in LUAD (a) and LUSQ (b). Top signaling pathways for genes showing increased expression are presented in red and those for genes showing decreased expression are presented in blue. (c, d) Running enrichment score with GO gene set of blood vessel morphogenesis, epidermis development, keratinocyte differentiation, and T cell activation signatures as the activated pathways in respective immune subtypes of LUAD (c) and LUSQ (d).
