# Supplementary material for: Tumor-infiltrating Leukocyte Profiling Defines Three Immune Subtypes of NSCLC with Distinct Signaling Pathways and Genetic Alterations
Source: Cancer Res Commun. 2023 Jun 13;3(6):1026–40. doi: 10.1158/2767-9764.CRC-22-0415 (PMC10263066; doi:10.1158/2767-9764.CRC-22-0415)
Supplement: Fig. S15 — Hazard ratios with deviation for specific signatures detected in the myeloid subtype of LUAD. The relationship between increased and decreased expression of signatures and event-free survival using transcriptome data from TCGA NSCLC was plotted. (a) hallmark gene set. (b) GO set. [file crc-22-0415-s15.pdf]

Fig. S15

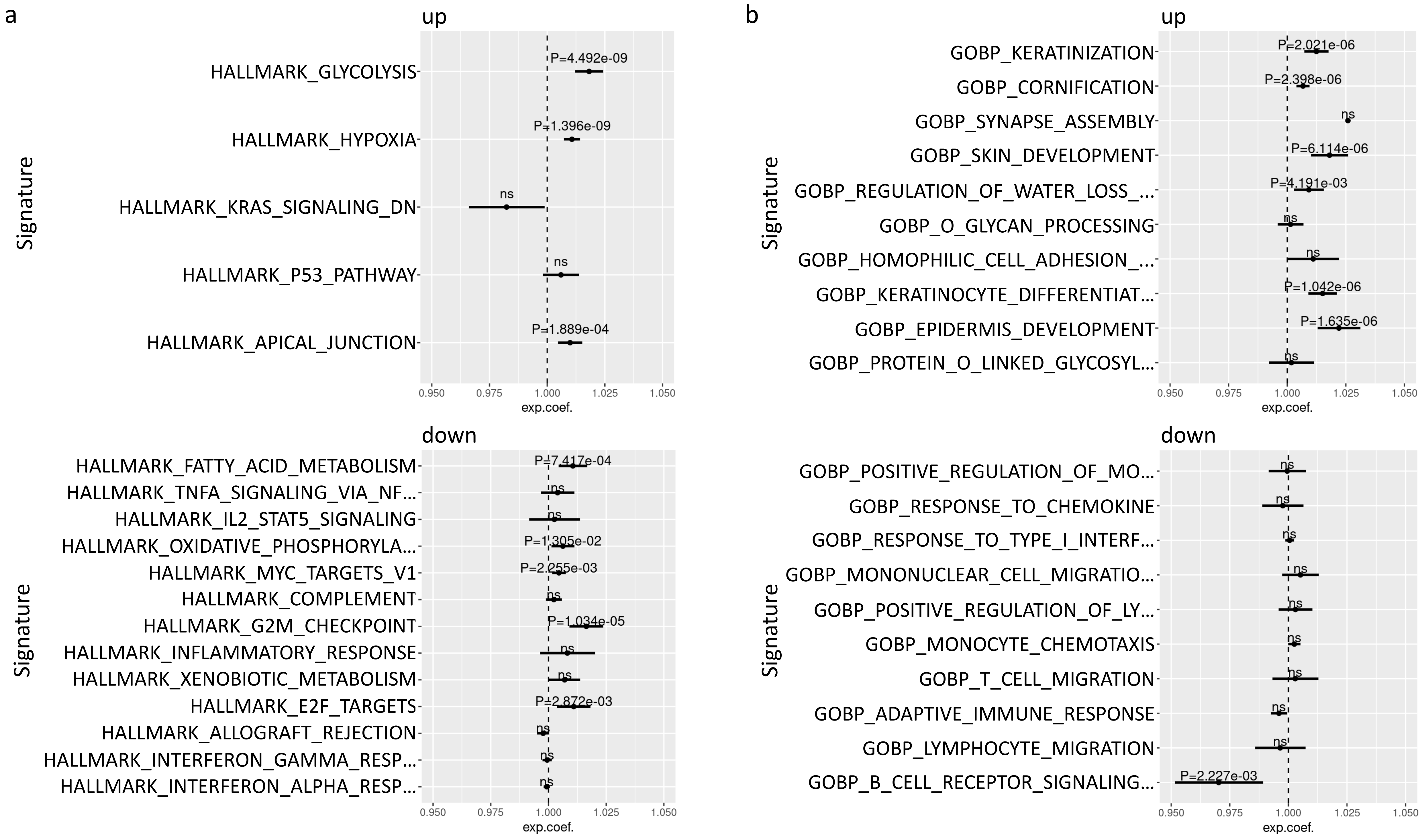

Figure S15. Hazard ratios with deviation for specific signatures detected in the myeloid subtype of LUAD. The relationship between increased and decreased expression of signatures and event-free survival using transcriptome data from TCGA NSCLC was plotted. (a) hallmark gene set. (b) GO set.
