# Supplementary material for: Tumor-infiltrating Leukocyte Profiling Defines Three Immune Subtypes of NSCLC with Distinct Signaling Pathways and Genetic Alterations
Source: Cancer Res Commun. 2023 Jun 13;3(6):1026–40. doi: 10.1158/2767-9764.CRC-22-0415 (PMC10263066; doi:10.1158/2767-9764.CRC-22-0415)
Supplement: Fig. S16 — Boxplot showing the total number of non-synonymous mutations in respective immune subtypes. Non-synonymous mutations were calculated from WES data. ns; not significant. [file crc-22-0415-s16.pdf]

Fig. S16

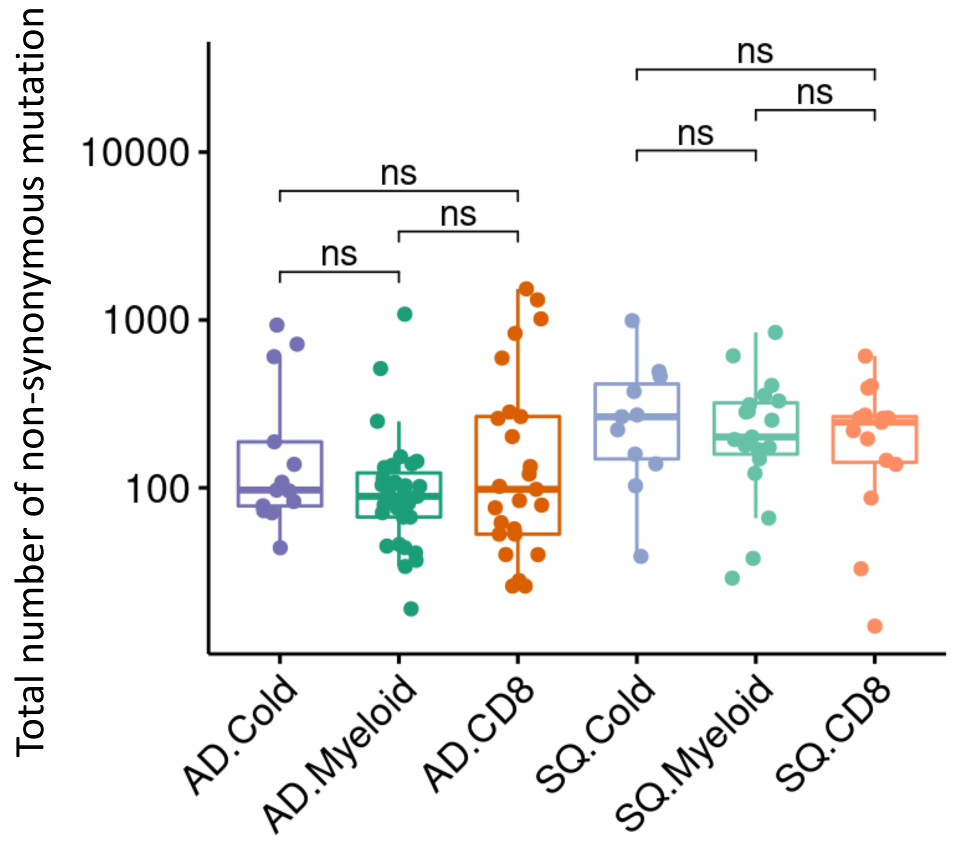

Figure S16. Boxplot showing the total number of non-synonymous mutations in respective immune subtypes. Non-synonymous mutations were calculated from WES data. ns; not significant.
