# Supplementary material for: Tumor-infiltrating Leukocyte Profiling Defines Three Immune Subtypes of NSCLC with Distinct Signaling Pathways and Genetic Alterations
Source: Cancer Res Commun. 2023 Jun 13;3(6):1026–40. doi: 10.1158/2767-9764.CRC-22-0415 (PMC10263066; doi:10.1158/2767-9764.CRC-22-0415)
Supplement: Fig. S17 — The relationship between TERT amplification and immune cell types in LUSQ. The correlations of the degree of TERT amplification with %CD45 of T, CD8+ T, mMDSC, and macrophage cells are plotted. [file crc-22-0415-s17.pdf]

Fig. S17

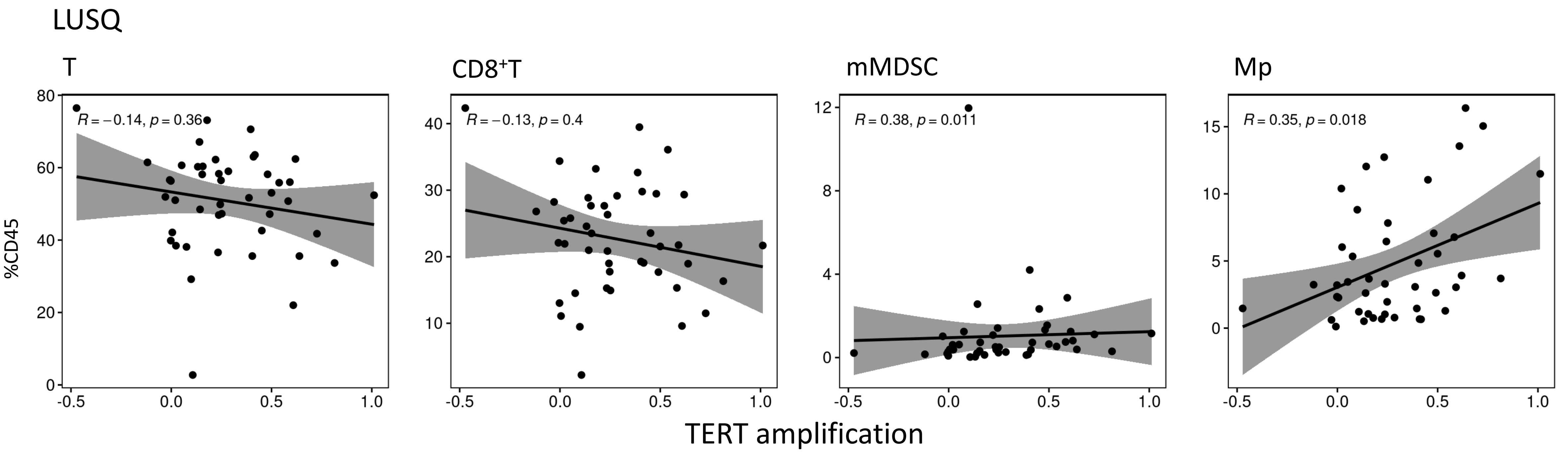

Figure S17. The relationship between *TERT* amplification and immune cell types in LUSQ. The correlations of the degree of *TERT* amplification with %CD45 of T, $CD8^+$ T, mMDSC, and macrophage cells are plotted.
