# Supplementary material for: Tumor-infiltrating Leukocyte Profiling Defines Three Immune Subtypes of NSCLC with Distinct Signaling Pathways and Genetic Alterations
Source: Cancer Res Commun. 2023 Jun 13;3(6):1026–40. doi: 10.1158/2767-9764.CRC-22-0415 (PMC10263066; doi:10.1158/2767-9764.CRC-22-0415)
Supplement: Table S1 — Patient characteristics [file crc-22-0415-s18.pdf]

Table S1

Patient characteristics

		No.（%）
Age（y）	Median (range)<br>＜65<br>≧65	70 (28-89)<br>89 (31.7)<br>192 (68.3)
Sex	Male<br>Female	185 (65.8)<br>96 (34.2)
Smoking history	Yes<br>No<br>NA	155 (79.1)<br>41 (20.9)<br>85
Pathological stage	0<br>I<br>II<br>III<br>IV	1 (0.4)<br>93 (33.1)<br>84 (29.9)<br>92 (32.7)<br>11 (3.9)
Histology	Adenocarcinoma<br>Squamous cell carcinoma<br>Pleomorphic carcinoma<br>Neuroendocrine carcinoma<br>Others	155 (55.2)<br>80 (28.5)<br>17 (6.1)<br>20 (7.1)<br>9 (3.2)
