# Supplementary material for: Tumor-infiltrating Leukocyte Profiling Defines Three Immune Subtypes of NSCLC with Distinct Signaling Pathways and Genetic Alterations
Source: Cancer Res Commun. 2023 Jun 13;3(6):1026–40. doi: 10.1158/2767-9764.CRC-22-0415 (PMC10263066; doi:10.1158/2767-9764.CRC-22-0415)
Supplement: Table S2 — Antibodies for FACS [file crc-22-0415-s19.pdf]

Table S2

Antibodies for FACS

Target antigen	Clone	Isotype	Label	Manufacturer	Cat. No.	RRID
CD16	3G8	mIgG1k	BUV805	BD	624287 (custom)	no RRID
CD152	BNI3	mIgG2ak	BUV737	BD	624286 (custom)	no RRID
HLA-DR	G46-6	mIgG2ak	BUV661	BD	565073	AB_2722500
CD86	2331	mIgG1k	BUV615	BD	624297 (custom)	no RRID
CD8	RPA-T8	mIgG1k	BUV563	BD	565695	AB_2744461
CD19	SJ25C1	mIgG1k	BUV496	BD	564655	AB_2744311
CD45RA	HI100	mIgG2bk	BUV395	BD	740298	AB_2740037
CD123	7G3	mIgG2ak	BV786	BD	564196	AB_2738662
CD11b	ICRF44	mIgG1k	BV750	BD	624380 (custom)	no RRID
CD68	Y1/82A	mIgG2bk	BV711	BD	565594	AB_2739297
CD56	NCAM16.2	mIgG2bk	BV650	BD	564057	AB_2738568
CD326	9C4	mIgG2bk	BV605	Biolegend	324224	AB_2562518
CD4	SK3	mIgG1k	BV570	BD	624298 (custom)	no RRID
L/D	-	-	BV510	invitrogen	L34965	no RRID
CD197	G043H7	mIgG2ak	BV421	Biolegend	353208	AB_11203894
CD11c	B-Ly6	mIgG1k	BB790	BD	624296 (custom)	no RRID
CD279	EH12.1	mIgG1k	BB700	BD	566460	AB_2744348
CD163	GHI/61	mIgG1k	BB660	BD	624295 (custom)	no RRID
CD3	SK7	mIgG1k	BB630	BD	624294 (custom)	no RRID
Ki67	B56	mIgG1k	A488	BD	558616	AB_647087
CD274	MIH1	mIgG1k	PE-Cy7	BD	558017	AB_396986
CD14	TuK4	mIgG2a	PE-Cy5.5	invitrogen	MHCD1418	AB_1464894
CD206	19.2/ 15-2	mIgG1k	PE-Cy5	BD/ Biolegend	551136/ 321108	AB_394066 /AB_571919
CD33	WM53	mIgG1k	PE-CF594	BD	562492	AB_2713912
FoxP3	206D	mIgG1k	PE	Biolegend	320108	AB_492986
CD45	2D1	mIgG1k	APC-H7	BD	560178	AB_1645479
