# Supplementary material for: Tumor-infiltrating Leukocyte Profiling Defines Three Immune Subtypes of NSCLC with Distinct Signaling Pathways and Genetic Alterations
Source: Cancer Res Commun. 2023 Jun 13;3(6):1026–40. doi: 10.1158/2767-9764.CRC-22-0415 (PMC10263066; doi:10.1158/2767-9764.CRC-22-0415)
Supplement: Table S3 — Antibodies for IHC [file crc-22-0415-s20.pdf]

Table S3

Antibodies for IHC

Primary antibody	Clone	Antigen retrieval	Dilution	Manufacturer	Detection system	Cat. No.	RRID
CD8	4B11	Citrate buffer	1:50	Novocastra	Envision	CD8-4B11-L-CE	AB_442068
CD20	L26	Citrate buffer	1:200	Thermo	Envision	MA5-13141	AB_10983209
FOXP3	236A/E7	Citrate buffer	1:100	Abcam	Envision with mouse LINKER	ab20034	AB_445284
Ki67	MIB1	Citrate buffer	1:100	Agilent	Envision	M724001-2	AB_2631211
CD33	EPR23051-101	Target retrieval solution, High pH	1:100	Abcam	Envision	ab269456	no RRID
